# Metabolic Reprogramming of Sulfur in Hepatocellular Carcinoma and Sulfane Sulfur-Triggered Anti-Cancer Strategy

**DOI:** 10.3389/fphar.2020.571143

**Published:** 2020-09-25

**Authors:** Ximing Zhang, Mianrong Chen, Xiang Ni, Yingying Wang, Xue Zheng, Hui Zhang, Shi Xu, Chun-tao Yang

**Affiliations:** ^1^ Affiliated Cancer Hospital & Institute of Guangzhou Medical University, Guangzhou Municipal and Guangdong Provincial Key Laboratory of Protein Modification and Degradation, School of Basic Medical Science, Guangzhou Medical University, Guangzhou, China; ^2^ Department of Chemistry, Brown University, Providence, RI, United States; ^3^ Department of Chemistry, Washington State University, Pullman, WA, United States

**Keywords:** bioinformatics, hepatocellular carcinoma, metabolic reprogramming, reactive sulfur species, sulfane sulfur

## Abstract

Metabolic reprogramming is a cancer hallmark. Although the reprogramming of central carbon has been well documented, the role of sulfur metabolism has been largely overlooked. Additionally, the effects of sulfur are sometimes contradictory in tumorigenesis. In this study, we aimed to investigate the gene expression profile in hepatocellular carcinoma (HCC) and the effects of reactive sulfur species (RSS) on HCC tumor cells. Furthermore, the cell imaging technology was applied to discover some potential anti-cancer compounds. Gene Set Enrichment Analysis (GSEA) of Gene Expression Omnibus (GEO) dataset (GSE102083) revealed that sulfur amino acid-related metabolism and vitamin B_6_ binding activity in HCC tissues were downregulated. Calculation of the interaction network identified nine hub genes, among which eight were validated by differential expression and survival analysis in the TCGA_LIHC cohort, and two (CSE and CBS) had the highest enrichment degree. The metabolomics analysis suggested that the hub genes were associated with RSS metabolism including H_2_S, H_2_S_2_, cystine, cysteine, homocysteine, cystathionine, and methionine. The cell viability assay demonstrated that H_2_S_2_ had significant anti-cancer effects in HCC SNU398 tumor cells. The cell imaging assay showed that treatment with H_2_S_2_ remarkably increased intracellular sulfane sulfur content. On this basis, the anti-cancer activity of some other sulfane sulfur compounds, such as DATS and DADS, was further verified. Lastly, according to the fact that HCC tumor cells preferentially take in cystine due to high expression of SLC7A11 (a cystine/glutamate transporter), persulfided cysteine precursor (PSCP) was tested for its sulfane sulfur release capability and found to selectively inhibit HCC tumor cell viability. Collectively, this study uncovered sulfur metabolism in HCC was reprogrammed, and provided a potential therapeutic strategy for HCC by donating sulfane sulfur.

## Introduction

In liver cancer, hepatocellular carcinoma (HCC) is the most common primary malignancy, which has been the leading cause of cancer-related death globally. Surgical resection was considered to be the standard and first option treatment of HCC, because of its resistance to radiotherapy or chemotherapy (Lohitesh et al., 2018). Unfortunately, many HCC patients may not have the chance of surgical resection because the diagnosis usually occurs at the late stage. Therefore, revealing the altered cell biological behavior with the gene expression profile at the early stage will contribute to the discovery of potential targets that are of therapeutic significance.

It has been known for decades that plants carrying organosulfur-containing compounds, like garlic, have anti-cancer effects (Weisberger and Pensky, 1957). Studies supported that many of garlic’s effects are attributed to the generation of sulfane sulfur, a reactive sulfur species (RSS) (Iciek et al., 2001; Iciek et al., 2012). For human beings, persulfide (RSSH), polysulfide (RSS_n_R), and thiosulfate (RSSO_3_) are major forms of sulfane sulfur in the body (Yuan et al., 2017; Akaike et al., 2017; Alvarez et al., 2017; Yang et al., 2019a; Liu H. et al., 2019). Additionally, some endogenous RSS, like cystine, cysteine, homocysteine, methionine, glutathione, and hydrogen sulfide (H_2_S), are not sulfane sulfur *per se*, but can be metabolized to produce sulfane sulfur (Jackson et al., 2012). However, the effects of these RSS on cancer therapy including HCC are contradictory, as both anti-cancer (Iciek et al., 2001; Iciek et al., 2012) and pro-cancer (Pan et al., 2014; Zhen et al., 2018; Yang et al., 2019b) activities have been reported, which hinders the sulfur-based HCC therapy.

Metabolic reprogramming is an important cancer hallmark that has been studied over a century but is still of research significance (DeBerardinis et al., 2008; Faubert et al., 2020; Zhou et al., 2020). In tumor cells, a variety of metabolic processes, such as aerobic glycolysis, glutamine catabolism, macromolecular synthesis, and redox homeostasis, are distinctively different from that in normal cells. This metabolic flexibility is believed to fuel tumor fast proliferation (Kim and DeBerardinis, 2019). However, the metabolism of RSS in HCC tissues has often been overlooked. Additionally, it is unclear whether the different therapeutic outcomes of sulfur compounds are associated with the reprogramed RSS metabolism. A recent study showed that the diet restriction of methionine (a sulfur amino acid) was able to inhibit tumor growth in colorectal cancer (Gao et al., 2019). On the other hand, the elevated level of homocysteine (Wu and Wu, 2002), or decreased level of vitamin B_6_ (Galluzzi et al., 2013), could promote tumorigenesis. As known, vitamin B_6_ (pyridoxal 5’-phosphate) is a cofactor of cystathionine-β-synthase (CBS) or cystathionine-γ-lyase (CSE/CTH) in the generation of RSS including H_2_S and H_2_S_2_ (Renga, 2011; Gregory et al., 2016). These studies inspired us to explore the overall difference between HCC and normal liver tissues in the RSS metabolism, by which some useful therapeutic strategies may be uncovered.

Transcriptomics analysis of high throughput RNA sequencing is a highly efficient means to discover the potential causes of unique HCC phenotype. Gene Set Enrichment Analysis (GSEA) is one of the most popular computational tools for transcriptomics analysis. Therefore, in this work, GSEA was used to demonstrate whether the sulfur metabolism was reprogrammed in HCC tissues. Then, through the bioinformatics analysis, the involved hub genes were revealed, their significance was verified, and the interacted RSS were discovered. With the cell imaging and viability assay, some RSS that have anti-HCC activity were suggested. Lastly, based on the enhanced cystine transport capacity in HCC tumor cells, persulfided cysteine precursor (PSCP) and a series of esterase-triggered sulfane sulfur donors were synthesized and their anti-HCC effects were evaluated.

## Materials and Methods

### Materials

Sodium sulfide (Na_2_S), sodium persulfide (Na_2_S_2_), sodium trisulfide (Na_2_S_3_), and Cell Counting Kit (CCK)-8 were purchased from Dojindo Lab (Kyushu, Japan). N-acetyl cysteine (NAC), cystine, cystathionine, and GSSG were bought from Thermo Fisher Scientific Co. (Pittsburgh, PA, USA). Gemcell™ fetal bovine serum (FBS) was supplied by Gemini Company (Woodland, CA, USA). SSP4, PSCP, and the controllable sulfane sulfur donors were synthesized following the literature procedure and their characterization matches reported data (Rietman et al., 1994; Chen et al., 2013; Artaud and Galardon, 2014).

### Cell Culture and Viability Assay

HCC (SNU387 and SNU398) tumor cells, lung adenocarcinoma A549 cells, prostate cancer PC3 cells, cervical cancer Hela cells, breast MDA-MB-231 cells, raw-blue macrophages, LO2 liver cells, and H9c2 cardiomyocytes were obtained from ATCC. The cells were maintained in DMEM-F12 medium supplemented with 10% FBS at 37°C under an atmosphere of 5% CO_2_ and 95% air. They were passaged and harvested with 0.25% trypsin.

Cell viability was measured with the CCK-8 kit. The cells were plated in 96-well plates at a density of 7,000 cells/well. When grown to approximately 60~70% confluence, they were treated correspondingly for 48 h. Then, the plates were washed and 100 μl of CCK-8 solution diluted with FBS-free medium was added. The cells were incubated for a further 2 h at 37°C. The absorbance was measured at 450 nm with a microplate reader (Molecular Devices, USA).

### Data Collection

The microarray data of four normal mouse liver tissues and four HCC tumor tissues were downloaded from Gene Expression Omnibus (GEO) (https://www.ncbi.nlm.nih.gov/geo/query/acc.cgi?acc=%20GSE102081) (Chiyonobu et al., 2018). For the Cancer Genome Atlas (TCGA) data, the analysis of differential expression was downloaded from GEPIA2 (http://gepia2.cancer-pku.cn/#analysis), and the survival data were downloaded from the Human Protein Atlas (https://www.proteinatlas.org/).

### Gene Set Enrichment Analysis

The expression dataset from GSE102081 was converted as follows: According to the probes of Agilent Sureprint G3ge8x60k in the first column, a column of gene symbol (mgi_symbol) was added. The subsequent columns were named C1, C2, C3, and C4 for the normal liver tissues, while H1, H2, H3, and H4 for the HCC tumor tissues. The data file was imported into Gene Set Enrichment Analysis (GSEA) 4.0.3 software for further calculation and illustration according to the protocol (Subramanian et al., 2005).

### Prediction of Hub Genes

The interaction of the leading-edge genes identified by GSEA was subjected to STRING analysis (https://string-db.org/) by constructing a protein-protein interaction (PPI) network. The disconnected nodes were not involved in this network. The data were downloaded and imported to Cytoscape 3.7 software for further calculation, visual analysis, and hub gene prediction.

### Observation of Intracellular Sulfane Sulfur Levels

HCC SNU398 tumor cells were inoculated in 24-well plates and grown to 60∼70% confluence. After treatment with the indicated RSS for 1 h, intracellular sulfane sulfur was measured with SSP4 fluorescent probe (Chen et al., 2013; Bibli et al., 2018). Briefly, the treated cells were incubated with 10 μM SSP4 in the presence of 20 μM cetyl trimethyl ammonium bromide (CTAB) in 1% FBS medium for 20 min in the dark. The cell imaging was carried out after a slight wash with PBS buffer. The intracellular fluorescence signal was visualized under the Nikon E600 Fluorescence microscope (Walpole, MA, US).

### Chemical Synthesis and Characterization

PSCP was synthesized following reported procedure (Rietman et al., 1994). NMR matches the reported data. ^1^H NMR (400 MHz, Methanol-*d4*) 4.24 (m, 1H), 3.93 (s, 3H), 3.44–3.47 (dd, 2H), 3.21–3.27 (m, 2H).

**Scheme 1:**
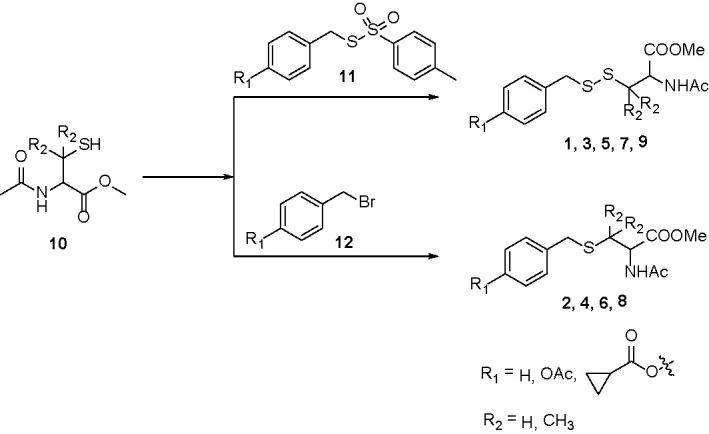
Synthesis of esterase-activated sulfane sulfur donors and their controls.

Compounds **1**, **3**, **5**, **7**, and **9** were synthesized by treating protected cysteine/penicillamine **(10)** with the corresponding tosylated para-substituted benzyl mercaptans (**11**). Briefly, to a solution of **10** (2 mM) in dichloromethane (10 ml) was added **11** (1.8 mM) and triethylamine (4 mM). Reaction was stirred under room temperature overnight. The mixture was then purified by column chromatography (**Scheme 1**).

Compounds **2**, **4**, **6**, and **8** were synthesized by (**10**) with the corresponding para-substituted benzyl bromides (**12**). Briefly, under argon, to a solution of **12** (1.9 mM) and triethylamine (5 mM) in dichloromethane (5 ml) was added a solution of **10** (2 mM) in dichloromethane dropwise. Reaction was stirred under argon overnight. The mixture was then purified by column chromatography (**Scheme 1**).

### Measurement of Sulfane Sulfur Content

SSP4 and CTAB were dissolved in DMSO and ethanol respectively to make 1 mM and 5 mM stock solutions. To initiate the experiment, 2 mM PSCP was prepared with PBS buffer (pH 7.4, 50 mM, 10 ml). At 5 min, 10 min, 20 min, 1 h, 1.5 h, 2 h, 4 h, 6 h, and 12 h, 80 µl of the above solution was aliquoted into a testing solution containing CTAB stock solution (80 µl), SSP4 stock solution (40 µl) and PBS buffer (pH 7.4, 50 mM, 3,800 µl), making the concentration of PSCP, SSP4, and CTAB 40, 10, and 100 µM, respectively. The resulting solution was incubated under room temperature for 10 min and fluorescence was analyzed by a fluorometer (Cary Eclipse, Agilent, USA).

### Statistical Analysis

The experiment data are presented as mean ± standard deviation (SD). Significance between groups was evaluated by one-way analysis of variance (ANOVA) followed by Student-Newman-Keuls Test using GraphPad Prism 8 software (SanDiego, US). A probability <0.05 was considered statistically significant.

## Result

### Sulfur Metabolism Is Reprogrammed in HCC Tissues

**Figure 1:**
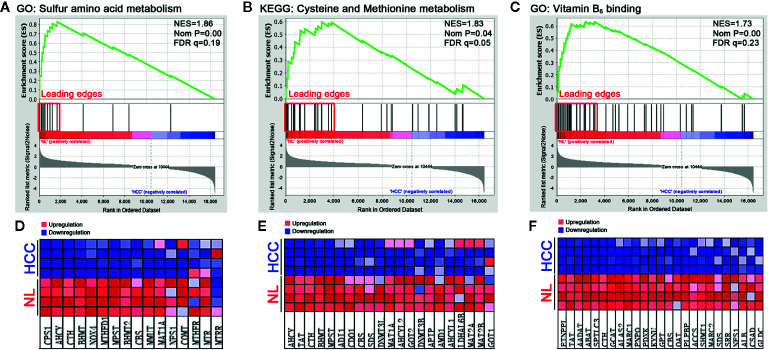
Functional enrichment analysis of genesets in HCC tumor tissues. A microarray dataset (GSE102081) of HCC tumor tissues and normal liver (NL) tissues was analyzed using the GSEA tool. **(A–C)** The enrichment indexes of three genesets are shown in **(A)** (sulfur amino acid metabolism), **(B)** (cysteine and methionine metabolism), and **(C)** (vitamin B_6_ binding activity), respectively. The genesets were considered to be significantly enriched at NES (normalized enrichment score) >1, Nom *P* (nominal p-value) <0.05, and FDR q-value <0.25. **(D–F)** Heatmaps of the enriched genes in **(A–C)** were created to display their relative expression.

To dissect the difference of sulfur metabolism between normal liver and HCC tumor tissues, the gene expression profile was screened using the GSEA tool. As presented in **Figure 1**, compared with the normal liver tissues, in HCC tumor tissues 12 out of 16 genes were negatively expressed in sulfur amino acid metabolism GO geneset (**Figures 1A, D**), 17 out of 32 genes were negatively expressed in cysteine and methionine metabolism KEGG geneset (**Figures 1B, E**), and 26 out of 48 genes were negatively expressed in vitamin B_6_ binding activity GO geneset (**Figures 1C, F**). The result indicates that sulfur-related metabolism is distinctively disturbed in the HCC phenotype.

### Prediction of Hub Genes in Leading-Edge Sets

**Figure 2:**
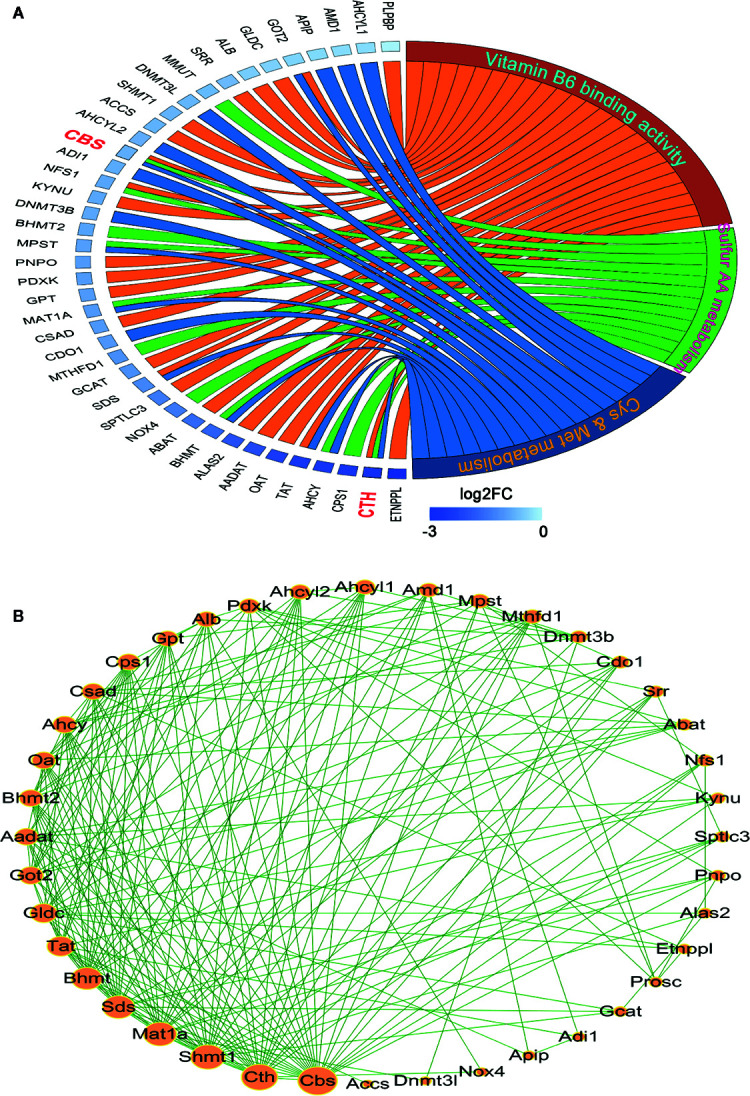
Analysis of the interaction among the leading-edge genes. **(A)** The ownership and relative expression of the leading-edge genes from three genesets (sulfur amino acid metabolism GO set, cysteine and methionine metabolism KEGG set, and vitamin B_6_ binding activity GO set) were presented through a chord diagram, which was drawn using the GOPlot R package. **(B)** The interaction of the genes was analyzed with the STRING online tool. The PPI network was constructed and the interaction degrees were calculated with Cytoscape 3.7 software.

To visualize the inter-relationships between these enriched genesets, a chord diagram was drawn. As shown in **Figure 2A**, 10 mutual genes were found, *i.e.* AHCY, BHMT, GOT2, MAT1A, MPST, NFS1, SDS, TAT, CBS, and CTH, among which CTH and CBS were shared by three genesets. Additionally, after the genes were sorted by expression level, which was displayed as log_2_ (Fold of Change), CTH was found to be dramatically downregulated.

In order to reveal the interaction of leading-edge genes, 12 enriched genes in sulfur amino acid metabolism GO set, 17 enriched genes in cysteine and methionine metabolism KEGG set, and 26 enriched genes in vitamin B_6_ binding activity GO geneset were subjected to the PPI analysis. As shown in **Figure 2B**, nine genes, *i.e.* CBS, CTH, SHMT1, MAT1A, SDS, BHMT, TAT, GLDC, and GOT2 had significantly high interaction degrees (>15), thereby being considered as hub genes responsible for the unique HCC phenotype.

### Expression and Significance of Hub Genes in HCC Patients

**Figure 3:**
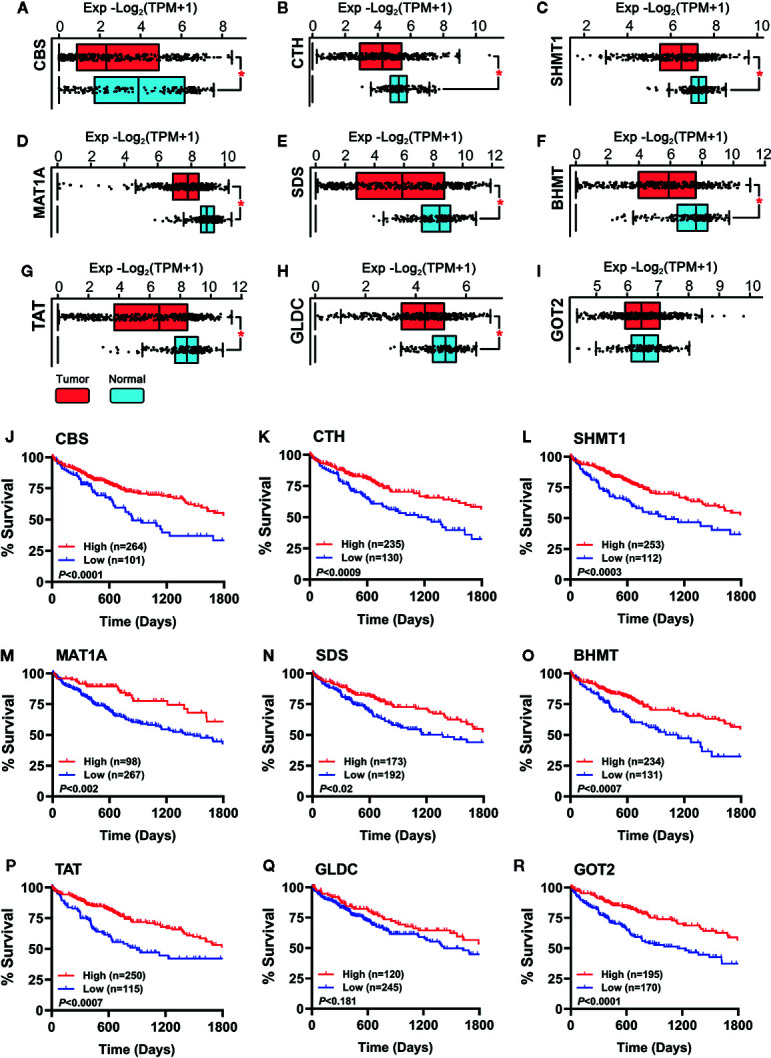
**(A–I)** Expression of the hub genes between 160 normal liver tissues (50 samples of TCGA_LIHC cohort and 110 samples in GTEx dataset) and 368 HCC tumor tissues of TCGA_LIHC cohort were observed. Data are shown as median ± quartile, *P < 0.001 *vs.* Normal liver tissues. **(J–R)** Analysis of the survival rate of HCC patients in TCGA_LIHC database between high expression (red lines) and low expression (blue lines) of the hub genes. The number of patients in either group and the Log-rank test’s *P* are shown in each panel.

To verify whether the hub genes were downregulated in HCC tumor tissues, the TCGA cohort (368 tumor samples *vs*. 50 normal liver samples) and GTEx dataset (110 normal liver samples) were applied for differential expression analysis. As shown in **Figures 3A–H**, all the hub genes except GOT2 (**Figure 3I**) were significantly downregulated in HCC tumor tissues compared with normal tissues (*P* < 0.001). Additionally, importance of the hub genes was evaluated by observing their influence on HCC patient survival in the TCGA cohort. As shown in **Figures 3J–R**, the downregulation of CBS, CTH, SHMT1, MAT1A, SDS, BHMT, TAT, and GOT2 could significantly reduce the survival rate, instead of GLDC (**Figure 3Q**). Therefore, GLDC was not involved in the following investigation.

### Identification of RSS That Interact With Hub Genes

**Figure 4:**
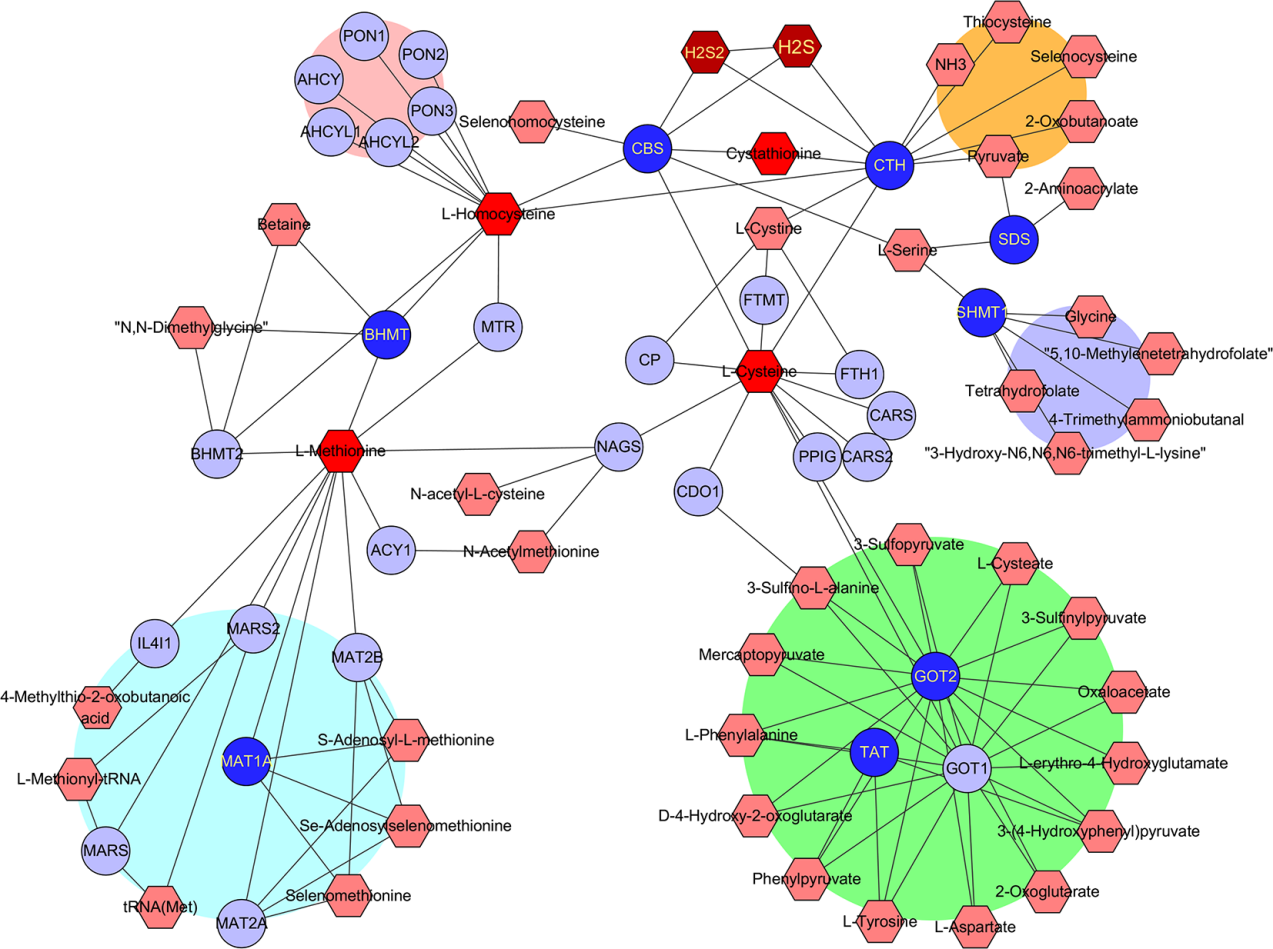
Identification of RSS affected by the hub genes. The hub genes and RSS compounds were imported into Metscape 3.0 software. The construction of network type was set as compound-gene, and the network was created. Hub genes (Dark blue ○), Related genes (Light blue ○), Interacted RSS compounds (Dark red hexagon).

To find out which RSS were potentially affected by the aforementioned hub genes, the metabolomics was investigated with the Metscape 3.0 tool, which is used to show the interaction of genes and compounds. As presented in **Figure 4**, the metabolism of methionine, cystine, cysteine, homocysteine, cystathionine, H_2_S, and H_2_S_2_ was found to be closely associated with the above hub genes. Therefore, the imbalance of these compounds is probably responsible for the development of HCC, and their application may affect the growth of HCC tumor cells.

### Effects of Identified RSS on Viability in HCC SNU398 Tumor Cells

**Figure 5:**
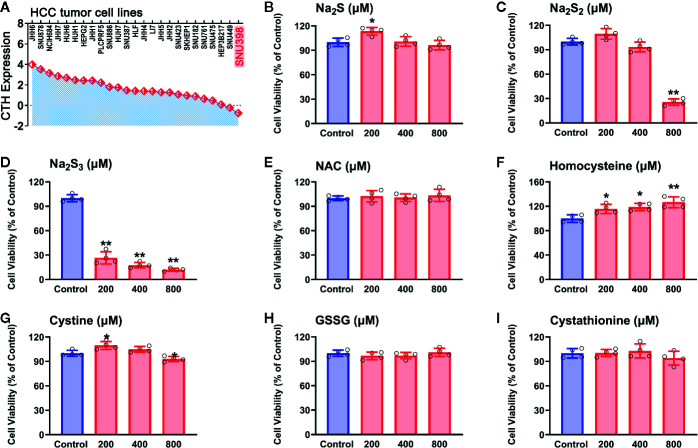
Effects of RSS on viability in HCC SNU398 tumor cells. **(A)** Expression of CTH in various common HCC liver cancer cell lines. **(B–I)** After treatment with increasing concentrations of Na_2_S **(B)**, Na_2_S_2_ **(C)**, Na_2_S_3_ **(D)**, NAC **(E)**, Homocysteine **(F)**, Cystine **(G)**, GSSG **(H)**, or Cystathionine **(I)** for 48 h, the cell viability was tested with CCK-8 assay. Data are expressed as mean ± SD of four independent experiments. ^*^ *P* < 0.05, ^**^ *P* < 0.01 *vs.* Control group.

With the identified RSS compounds that can interact with the hub genes, we investigated their influence on the viability in HCC SNU398 tumor cells. This cell line has relatively low CTH expression among 25 HCC tumor cell lines (**Figure 5A**), which is consistent with the discovery of reprogrammed sulfur metabolism in HCC tissues. Therefore, SNU398 tumor cells were applied in the following experiments. As shown in **Figure 5**, comparing with H_2_S (in the form of Na_2_S) (**Figure 5B**), treatment of the cells with H_2_S_2_ (in the form of Na_2_S_2_) (**Figure 5C**) or H_2_S_3_ (in the form of Na_2_S_3_) (**Figure 5D**) for 48 h remarkably decreased SNU398 cell viability, and the effect of H_2_S_3_ was much stronger. On the contrary, treatment with 200 μM H_2_S increased cell viability (**Figure 5B**). Additionally, RSS, such as sulfur-containing amino acids (NAC, homocysteine, and cystine), as well as derived peptide (cystathionine and GSSG), did not show inhibitory effects on the cell viability (**Figures 5E–I**).

**Figure 6:**
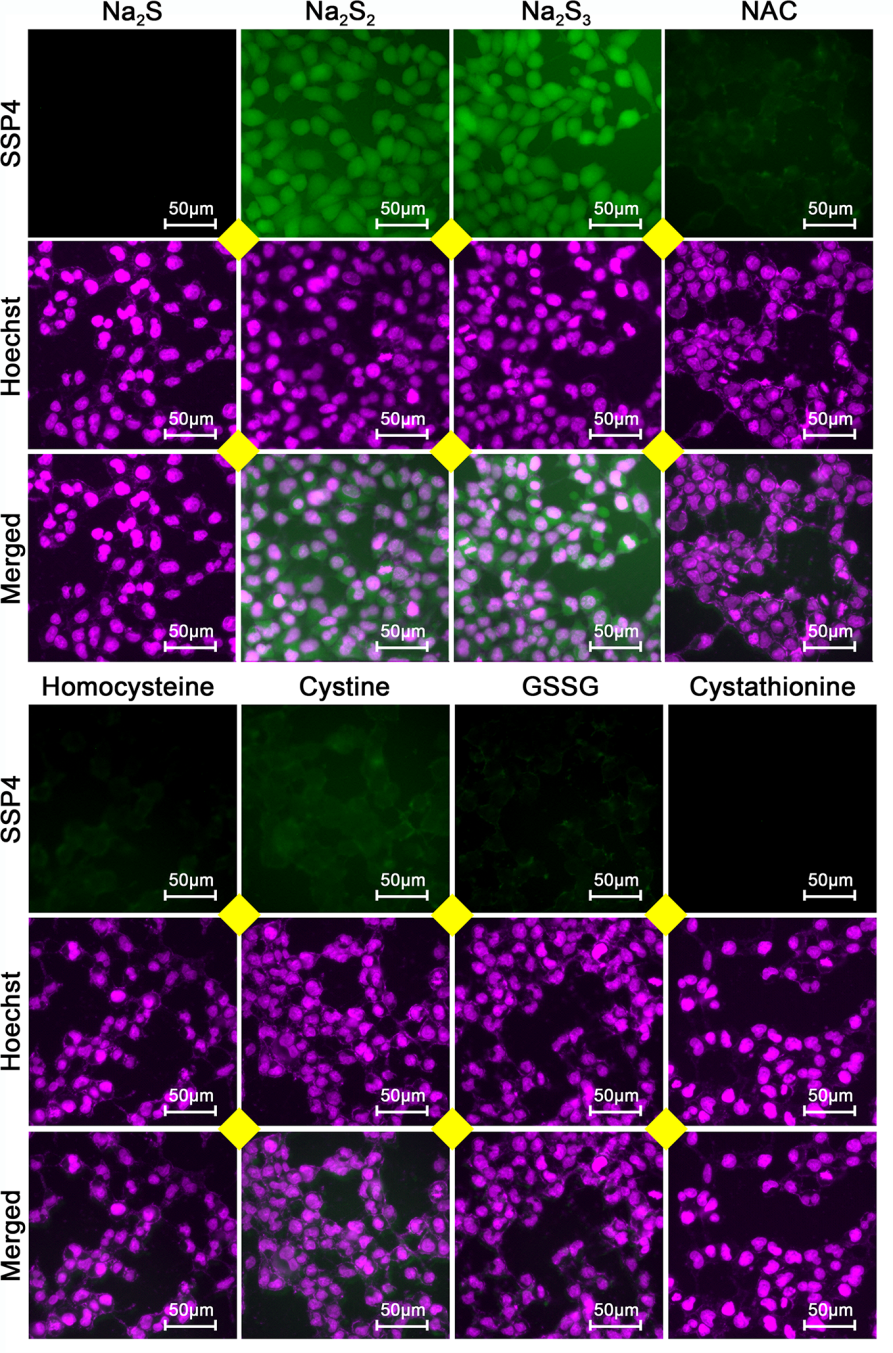
Effects of RSS on intracellular sulfane sulfur content in HCC SNU398 cells. After treatment with 400 μM of Na_2_S, Na_2_S_2_, Na_2_S_3_, NAC, Homocysteine, Cystine, GSSG, or Cystathionine for 1 h, the intercellular sulfane sulfur was observed with fluorescent probe SSP4 staining followed by photofluorography, and the cell nuclei were labeled through Hoechst 33324 staining.

To examine whether the effects of H_2_S_2_ and H_2_S_3_ were associated with reactive sulfane sulfur, the cell imaging technology was applied to observe intracellular sulfane sulfur content. As shown in **Figure 6**, the treatment with H_2_S_2_ or H_2_S_3_ could clearly enhance the intracellular sulfane sulfur content, while other RSS compounds did not show significant effects (**Figure 6**). Moreover, scavenging sulfane sulfur with excess GSH remarkably attenuated Na_2_S_3_-induced anti-HCC effects (**Supplementary Figure 1**). The data indicate that among RSS, sulfane sulfur may be important for HCC therapy.

### Sulfane Sulfur Mediates RSS-Induced Anti-HCC Effects in SNU398 Tumor Cells

**Figure 7:**
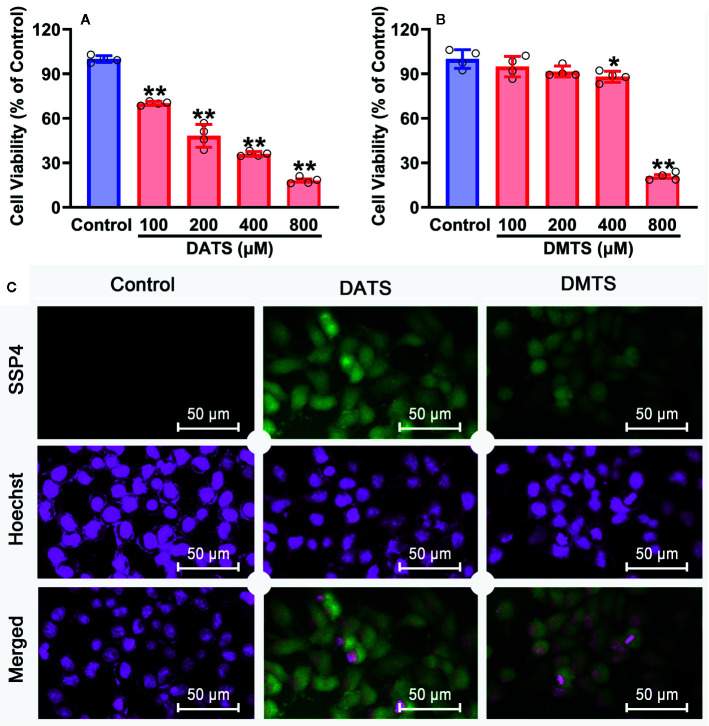
Anti-cancer effects of sulfane sulfurs in HCC SNU398 cells. **(A**, **B)** The cells were treated with increasing concentrations of DATS or DMTS for 48 h. The cell viability was measured with CCK-8 assay. Data are expressed as mean ± SD of four independent experiments. ^*^ *P* < 0.05, ^**^ *P* < 0.01 *vs*. Control group. **(C)** After the treatment with 400 μM DATS or DMTS, the intercellular content of sulfane sulfur was observed with SSP4 staining followed by photofluorography, and the cell nuclei were labeled using Hoechst 33324.

Since H_2_S_2_ and H_2_S_3_ could reduce SNU398 cell viability and increase intracellular sulfane sulfur content, and scavenging sulfane sulfur could attenuate H_2_S_3_-induced anti-HCC effects, we further validated this finding by sulfane sulfur-containing compounds. As shown in **Figure 7A**, exposure of SNU398 cells to diallyl trisulfide (DATS) significantly reduced cell viability. Such inhibitory effects were also observed in dimethyl trisulfide (DMTS), although only under high concentrations (**Figure 7B**). Additionally, the treatment with DATS or DMTS could increase intracellular sulfane sulfur contents (**Figure 7C**). The result supports the significance of sulfane sulfur in HCC therapy.

### Design, Synthesis, and Evaluation of Controllable Sulfane Sulfur Donors

**Figure 8:**
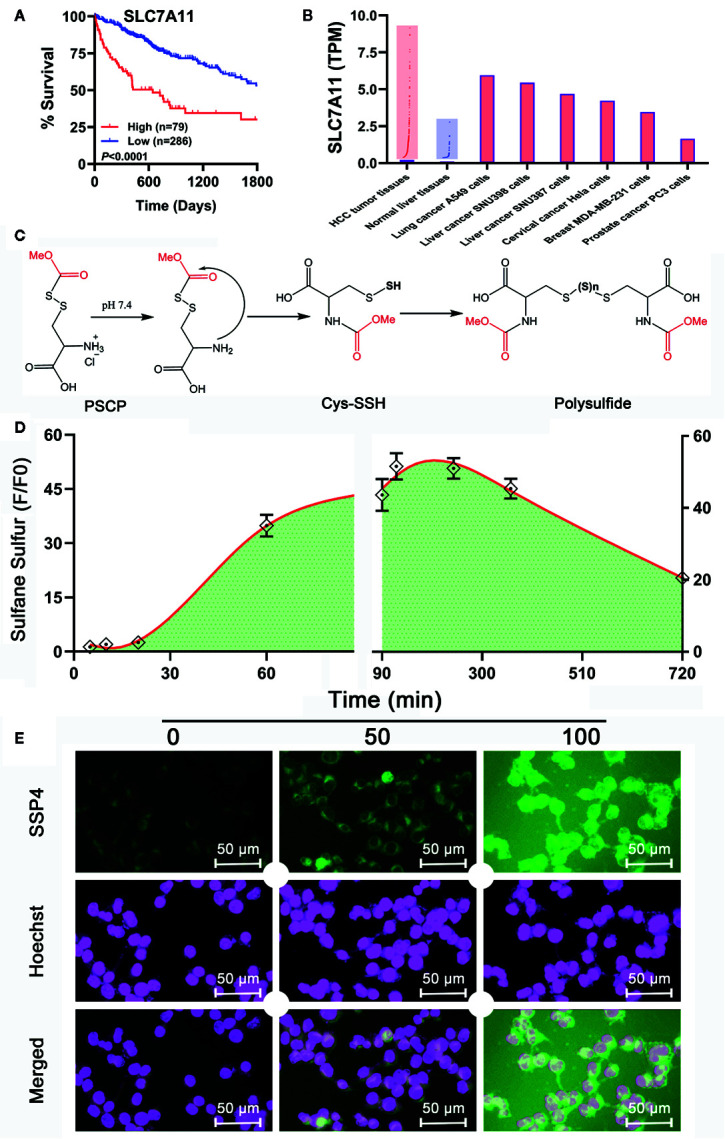
Development of a sulfane sulfur donor-specific to tumor tissues. **(A)** With TCGA cohort, effects of SLC7A11 on HCC patient survival between high (red line) and low (blue line) expression groups were examined using Log-rank test. **(B)** Analysis of TCGA cohort and Cancer Cell Line Encyclopedia for SLC7A11 expression in HCC patient tissues and tumor cell lines, respectively. **(C)** Mechanism underlying sulfane sulfur generation from PSCP. **(D)** The generated sulfane sulfur in PBS buffer was measured with a spectrophotometer. **(E)** After treatment of SNU398 cells with 200 μM PSCP for different times, intracellular sulfane sulfur was observed with SSP4 staining followed by photofluorography, and the cell nuclei were labeled using Hoechst 33324 staining.

Although Na_2_S_2_ and Na_2_S_3_ could inhibit the viability of SNU398 cells by producing sulfane sulfur, their usage probably has some problems. First, they are difficult to handle due to fast decomposition. Additionally, they may contain impurities like Na_2_S, further complicating the situation. Especially, Na_2_S_2_ and Na_2_S_3_ have limited trans-membrane ability. In the TCGA dataset, SLC7A11 was found to be a pivotal risk for the poor survival of HCC patients (**Figure 8A**). This gene is highly expressed in HCC tumor tissues, as well as some tumor cell strains including SNU398 and SNU387 liver cancer cells (**Figure 8B**). Since SLC7A11 protein is responsible for the transport of cystine or cysteine, we envisioned sulfane sulfur donors with a structure similar to cystine/cysteine might have better efficiency for HCC’s therapy. Additionally, they would be more stable and flexible due to the absence of free persulfide (R-SSH). As such, we prepared and tested S-(methoxycarbonylsulfenyl) cysteine hydrochloride, a persulfided cysteine precursor (PSCP). Under physiological pH, PSCP can undergo an intramolecular acyl transfer reaction to release cysteine persulfide (Cys-SSH), which eventually degrades to form polysulfides (**Figure 8C**), similar to a known design (Artaud and Galardon, 2014). Additionally, mass spectroscopy identified cysteine di-, tri-, and tetrasulfide derivatives in the solution (**Supplementary Figure 2**). These compounds are not only evidence of the persulfide formation, but also are sulfane sulfur themselves for tri- and tetrasulfide. Both persulfides and polysulfides belong to sulfane sulfur, so PSCP can be considered as the precursor of cysteine-derived sulfane sulfur. As expected, under physiological pH, sulfane sulfur formed from PSCP (2 mM) reached the maximum concentration in 120 min. Significant amounts of sulfane sulfur were still detectable after 720 min (**Figure 8D**). The cell imaging assay showed that treatment with PSCP was able to increase intracellular sulfane sulfur contents in SNU 398 cells (**Figure 8E**).

**Figure 9:**
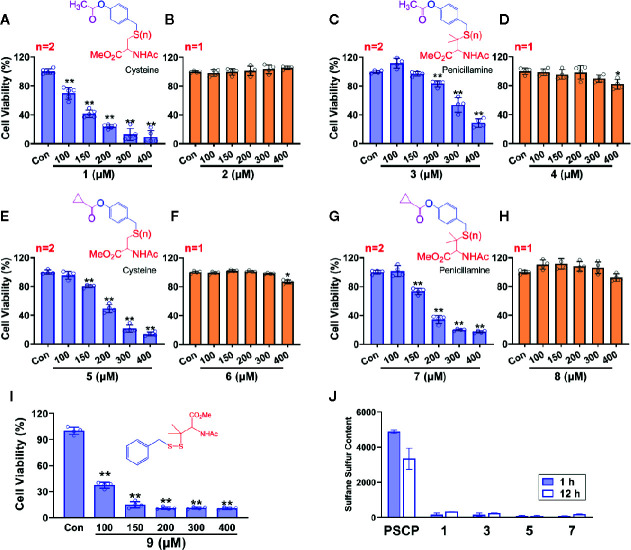
Controllable sulfane sulfur compounds-mediated inhibition of cell viability. HCC SNU398 cells were treated with esterase-triggered sulfane sulfur donor 1 **(A)**, 3 **(C)**, 5 **(E)**, or 7 **(G)**, their control compounds 2 **(B)**, 4 **(D)**, 6 **(F)**, or 8 **(H)**, as well as 9 **(I)**, which does not generate sulfane sulfur. After 48 h, the cell viability was tested with CCK-8 assay. **(J)** Sulfane sulfur generated from the **1**, **3**, **5**, and **7** was measured at 1 and 12 h with a fluorescence probe SSP4. Data are expressed as mean ± SD of four independent experiments. *P < 0.05, ^**^ *P* < 0.01 *vs*. Control group.

However, as found in **Figures 8C, D** and **Supplementary Figure 1**, the generation of sulfane sulfur from PSCP was spontaneous and the effect of sulfane sulfur was dramatically attenuated by GSH, which may impede its future application. Therefore, we have attempted to develop donors triggered by physiological stimuli, like enzymes. As such, four esterase-triggered donors were designed and synthesized (**1**, **3**, **5**, and **7**), which consisted of a cleavable ester trigger, a self-immolative linker, and a sulfane sulfur-releasing moiety. These donors can theoretically release protected cysteine or penicillamine persulfide, a form of sulfane sulfur, upon reaction with intracellular esterase. Meanwhile, four control compounds (**2**, **4**, **6**, and **8)**, which should only release protected cysteine/penicillamine, were also prepared. The characterizations of Compound 1-8 can be found in **Supplementary Figure 3**. Additionally, we have made a compound, which has no ester trigger and is therefore not able to release persulfide **(9)**. As shown in **Figure 9**, treatment with these esterase-triggered donors (**1**, **3**, **5**, and **7**) significantly inhibited SNU398 cell viability (**Figures 9A, C, E, G**), while no obvious inhibitory effects in the control compounds (**2**, **4**, **6**, and **8)** was found (**Figures 9B, D, F, H**). Although these results are promising, compound **9**, which theoretically cannot generate sulfane sulfur, also reduced cell viability. Additionally, we were not able to detect sulfane sulfur generation from these donors using SSP4. The data suggest that inhibitory effects of (**1**, **3**, **5**, and **7**) may not come from sulfane sulfur. Although it is still not clear how these compounds achieved inhibitor effects, the present design of esterase-triggered sulfane sulfur donor should be improved.

### Evaluation of PSCP’s Anti-Cancer Effects in Various Cells

**Figure 10:**
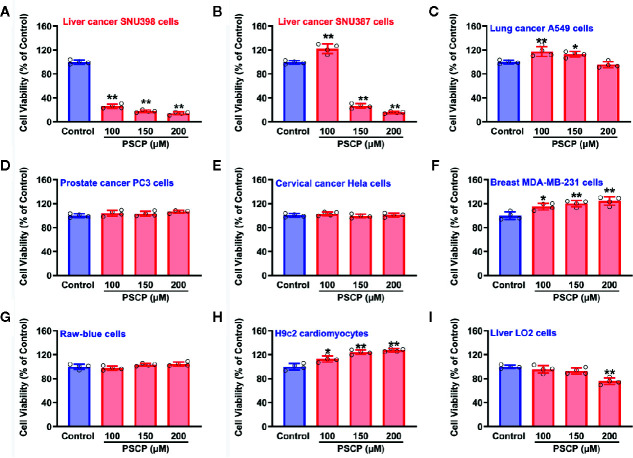
Effects of PSCP on viability in various cell strains. **(A)** Liver cancer SNU398 cells, **(B)** Liver cancer SNU387 cells, **(C)** Lung cancer A549 cells, **(D)** Prostate cancer PC3 cells, **(E)** Cervical cancer Hela cells, **(F)** Breast MDA-MB-231 cells, **(G)** Raw-blue macrophages, **(H)** H9c2 cardiomyocytes, and **(I)** Liver LO2 cells. Different types of cell strains were treated with increasing concentrations of PSCP for 48 h. The cell viability was tested with CCK-8 assay. Data are expressed as mean ± SD of four independent experiments. ^*^ *P* < 0.05, ^**^ *P* < 0.01 *vs.* Control group.

As shown in **Figure 10**, in HCC SNU398 cells (**Figure 10A**) and another liver cancer cell line SNU387 (**Figure 10B**), PSCP could exert obvious anti-cancer effects. However, in other tumor cell strains, including lung cancer A549 cells (**Figure 10C**), prostate cancer PC3 cells (**Figure 10D**), cervical cancer Hela cells (**Figure 10E**), and breast MDA-MB-231 cells (**Figure 10F**), remarkable anti-cancer effects were not observed. Notably, in normal cells, such as Raw-blue macrophages, H9c2 cardiomyocytes, as well as normal liver cells, PSCP did not show severe toxicity. The result suggests that sulfane sulfur (PSCP) is able to inhibit tumor cell viability in a liver cancer-specific manner.

## Discussion

Metabolic reprogramming is a distinctive hallmark of cancer. The reprogrammed glucose metabolism in tumorigenesis has been studied for many years (Kato et al., 2018; Lin et al., 2019). Additionally, the metabolism of amino acids, such as cysteine, homocysteine, glutamine, and methionine, are attracting increased attention in recent years (Gao et al., 2019; Lieu et al., 2020). Through metabolic reprogramming, tumor cells can generate large amounts of acids, reduced compounds, as well as one-carbon units, to support their rapid growth (Pavlova and Thompson, 2016; Sun et al., 2020).

Sulfur metabolism in HCC tumor tissues is still a novel field of research. In this work, we observed the sulfur metabolism was reprogrammed in HCC through the bioinformatic GSEA screening. The scanning revealed that the gene sets of sulfur amino acid metabolism, cysteine and methionine metabolism, and vitamin B_6_ binding activity were remarkably impaired in HCC tissues. The further PPI analysis suggested that CBS, CTH, SHMT1, MAT1A, SDS, BHMT, TAT, and GOT2 were significant hub genes. Importantly, the TCGA analysis validated their significance in HCC patients. Through these data, we believe that the genes related to sulfur metabolism should be dysregulated in HCC tissues, which may be responsible for the low survival of patients. Recently, some studies demonstrated that high levels of methionine and homocysteine were able to promote tumorigenesis (Sun et al., 2002; Gao et al., 2019), while the methionine-restricted diet arrested tumor growth and increased chemotherapy sensitivity (Hoffman, 2019). Moreover, a previous study showed that the content of vitamin B_6_ pyridoxal phosphate and the activity of pyridoxine kinase in Morris hepatomas were lower than those in normal liver tissues (Meisler et al., 1982). The antioxidant NAC, a cysteine equivalent, was reported to accelerate lung cancer progression (Sayin et al., 2014). These studies support that the increased methionine, homocysteine, and cysteine, as well as the decreased vitamin B_6_ binding activity, are probably important causes for HCC tumor growth, which is consistent with the present analyses. For this reason, metabonomic analysis can bring to light the potential interaction between RSS and the hub genes in HCC tissues. Meanwhile, the findings provide a reasonable explanation that through sulfur metabolic reprogramming, HCC tumor cells can raise the content of methionine, homocysteine, or cysteine to fuel their fast proliferation.

Reprogrammed sulfur metabolism is likely to make HCC tumor cells adapt to a new sulfur environment. Since the metabolism of RSS was impaired, we scanned the effects of RSS on HCC tumor cell viability. It was found that Na_2_S_2_ and Na_2_S_3_ remarkably inhibited cell viability, while the other RSS compounds did not exert such effects. It is worth noting that Na_2_S_2_ and Na_2_S_3_ are sulfane sulfur donors (Jiang et al., 2019). Also, DATS and DMTS belong to sulfane sulfurs, which could also attenuate HCC tumor cell viability in the present study, consistent with the previous reports (Weisberger and Pensky, 1957; Liu X. et al., 2019). Some researchers considered that anti-cancer effects from garlic-derived compounds were attributed to H_2_S generation. However, in this study, we did not observe that H_2_S had anti-cancer effects at the same treatment profile. In recent years, the studies of H_2_S on HCC tumorigenesis are conflicting, *i.e.* anti-cancer *vs*. pro-cancer (Wu et al., 2017; Yang et al., 2019b; Wu et al., 2019). Those studies on H_2_S’s anti-HCC activity, we think, are probably due to the generation of sulfane sulfur from Na_2_S/NaHS.

To support the above hypothesis, the cell imaging assay was performed and the results demonstrated that the treatment with Na_2_S_2_ or Na_2_S_3_ was able to enhance the intracellular sulfane sulfur levels, so did DATS and DMTS. Importantly, their treatment all showed anti-HCC effects. Moreover, scavenging of sulfane sulfur with excess GSH significantly attenuated sulfane sulfur-induced anti-HCC effects. These findings support the notion that the application of sulfane sulfurs may be an effective therapeutic strategy, especially for the HCC tumor cells with reprogrammed sulfur metabolism.

Notably, Na_2_S_2_ and Na_2_S_3_ are unstable chemicals, while commercial DATS is highly volatile, which will hinder their applications. Through the TCGA cohort analysis, it was shown that SLC7A11 (xCT) is upregulated in HCC tissues, indicating an increased cystine transportability. We then synthesized PSCP and found it was able to produce cystine sulfane sulfurs including per-, tri-, and tetrasulfide. The continuous monitoring indicated that the content of sulfane sulfur could keep stable for at least ten hours in aqueous solution. Additional cell experiments showed that the treatment with PSCP did not only enhance intracellular sulfane sulfur, but also inhibited cell viability. Furthermore, we tested the effects of PSCP in other cancer cells like lung cancer A549 cells, prostate cancer PC3 cells, cervical cancer Hela cells, and breast MDA-MB-231 cells, and did not find remarkable inhibitory activity. We surmise that the sulfur metabolism may not be reprogrammed, although some of these cells have high cystine transportability, for example the increased SLC7A11 expression in A549 cells. Interestingly, in H9c2 cardiomyocytes and normal liver LO2 cells, the cytotoxicity was not very high, indicating its safety for the future *in vivo* application. Lastly, because of the spontaneous release of sulfane sulfur from PSCP and the influence of GSH, we synthesized esterase-triggered sulfane sulfur donors. Although the donors may produce sulfane sulfur theoretically and showed promising inhibition on cell viability, we were not able to observe the generation of sulfane sulfur from them. Therefore, the present design of enzyme-triggered sulfane sulfur donors should be improved in the future.

In conclusion, we have discovered that sulfur-related metabolism in HCC is reprogrammed. The RSS screening indicates that HCC tumor cells are sensitive to sulfane sulfur. The selective inhibition of HCC cell viability from PSCP, a sulfane sulfur donor, further validates this finding. This study provides a basic evidence for the treatment of HCC that has sulfur metabolic reprogramming.

## Data Availability Statement

All datasets presented in this study are included in the article/**Supplementary Material**.

## Author Contributions

CY, SX, and XMZ designed the experiments and wrote the manuscript. MC, XN, YW, and HZ performed all the experiments and statistical analyses. All authors contributed to the article and approved the submitted version.

## Funding

This work was supported by Natural Science Foundation of Guangdong Province (2017A030313892) and Guangzhou Key Medical Discipline Construction Project.

## Conflict of Interest

The authors declare that the research was conducted in the absence of any commercial or financial relationships that could be construed as a potential conflict of interest.
