# Supplementary figures and images for: Metabolic Reprogramming of Sulfur in Hepatocellular Carcinoma and Sulfane Sulfur-Triggered Anti-Cancer Strategy
Source: Front Pharmacol. 2020 Sep 25;11:571143. doi: 10.3389/fphar.2020.571143 (PMC7556288; doi:10.3389/fphar.2020.571143)

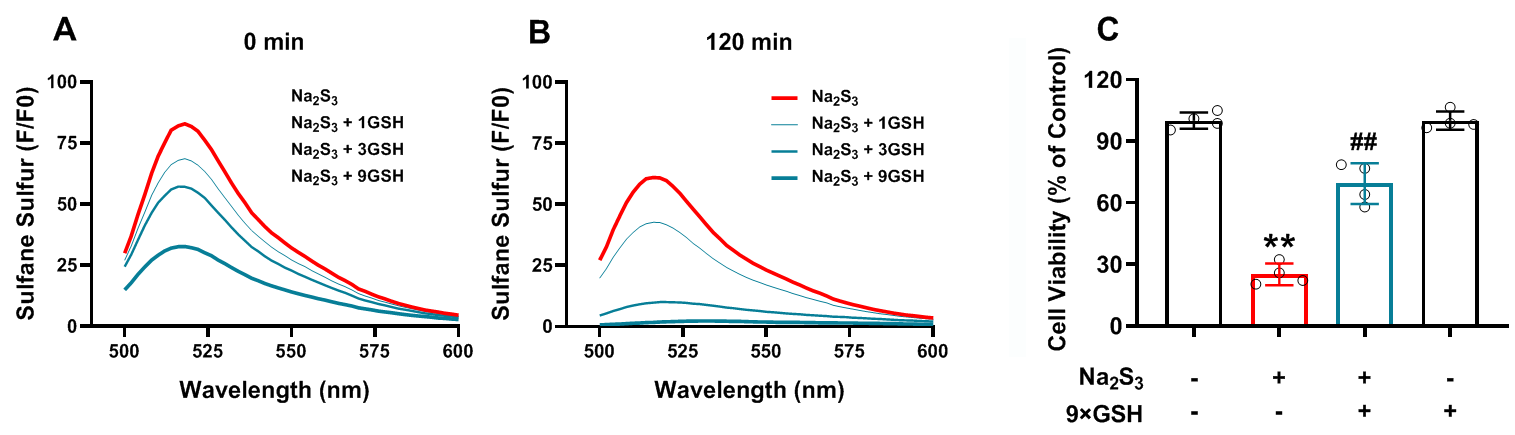

Supplement: Supplementary file 2 [file Image_1.tif]
