# Supplementary material for: Metabolic Reprogramming of Sulfur in Hepatocellular Carcinoma and Sulfane Sulfur-Triggered Anti-Cancer Strategy
Source: Front Pharmacol. 2020 Sep 25;11:571143. doi: 10.3389/fphar.2020.571143 (PMC7556288; doi:10.3389/fphar.2020.571143)
Supplement: Supplementary file 3 [file Image_2.pdf]

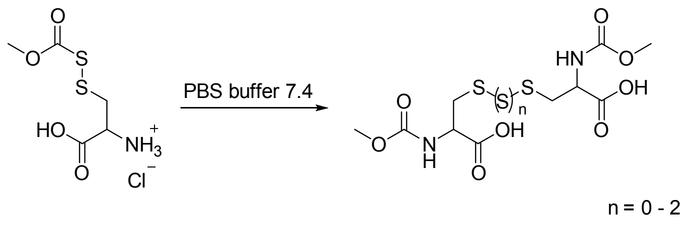

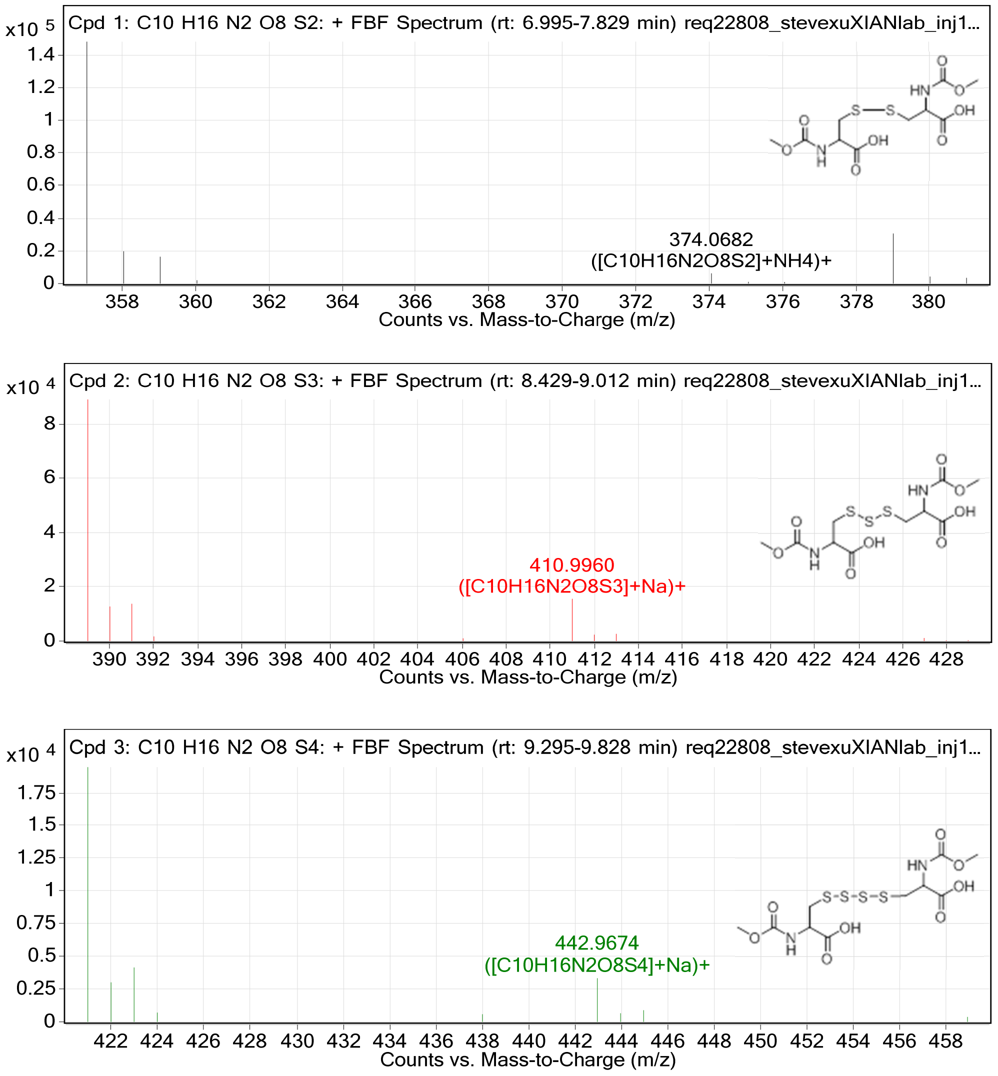

Supplementary Figure 2 PSCP (2 mM) was dissolved in buffer (PBS, 7.4) and incubated under room temperature for 6 h. The resultant mixture was directly analyzed by MS spectropscopy.
