# Supplementary material for: Metabolic Reprogramming of Sulfur in Hepatocellular Carcinoma and Sulfane Sulfur-Triggered Anti-Cancer Strategy
Source: Front Pharmacol. 2020 Sep 25;11:571143. doi: 10.3389/fphar.2020.571143 (PMC7556288; doi:10.3389/fphar.2020.571143)
Supplement: Supplementary file 4 [file Image_3.pdf]

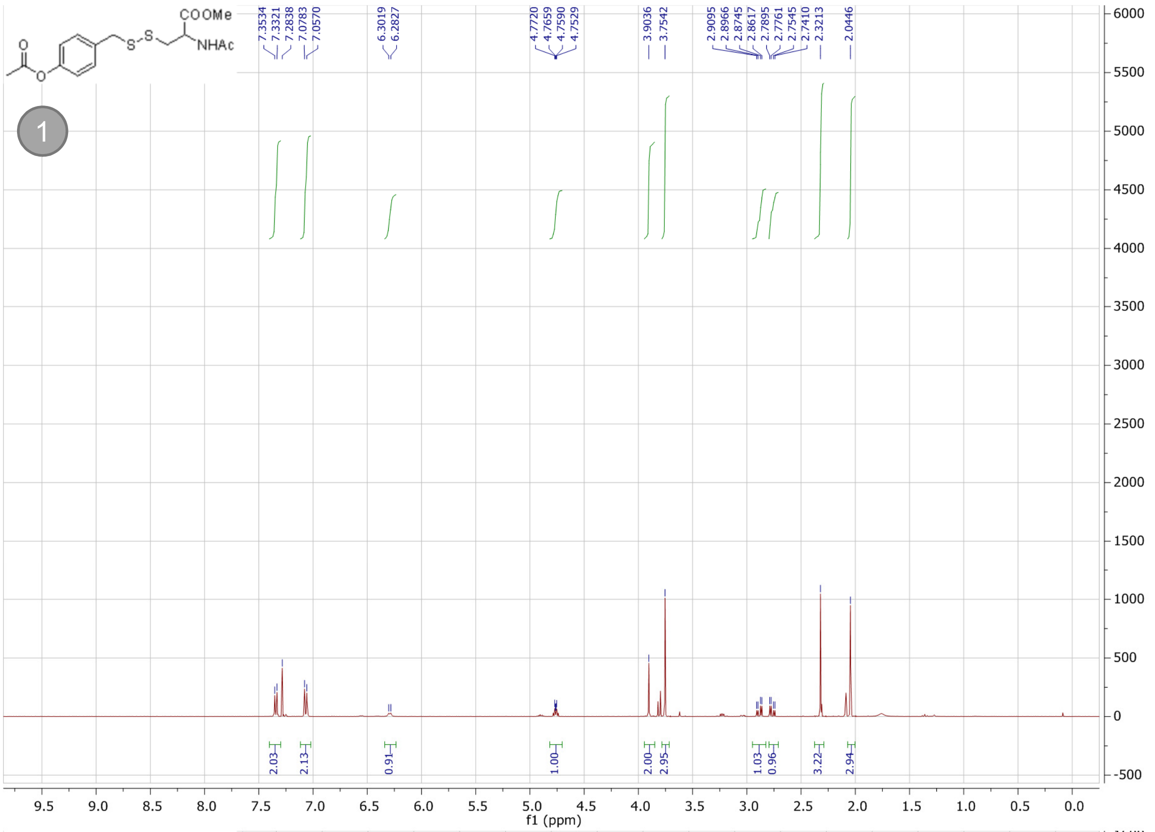
COOMe
NHAc
S
S
O
O
1
7.3534
7.3321
7.2838
7.0783
7.0570
6.3019
6.2827
4.7720
4.7659
4.7590
4.7529
3.9036
3.7542
2.9095
2.8966
2.8745
2.8617
2.7895
2.7761
2.7545
2.7410
2.3213
2.0446
2.03
2.13
0.91
1.00
2.00
2.95
1.03
0.96
3.22
2.94
f1 (ppm)

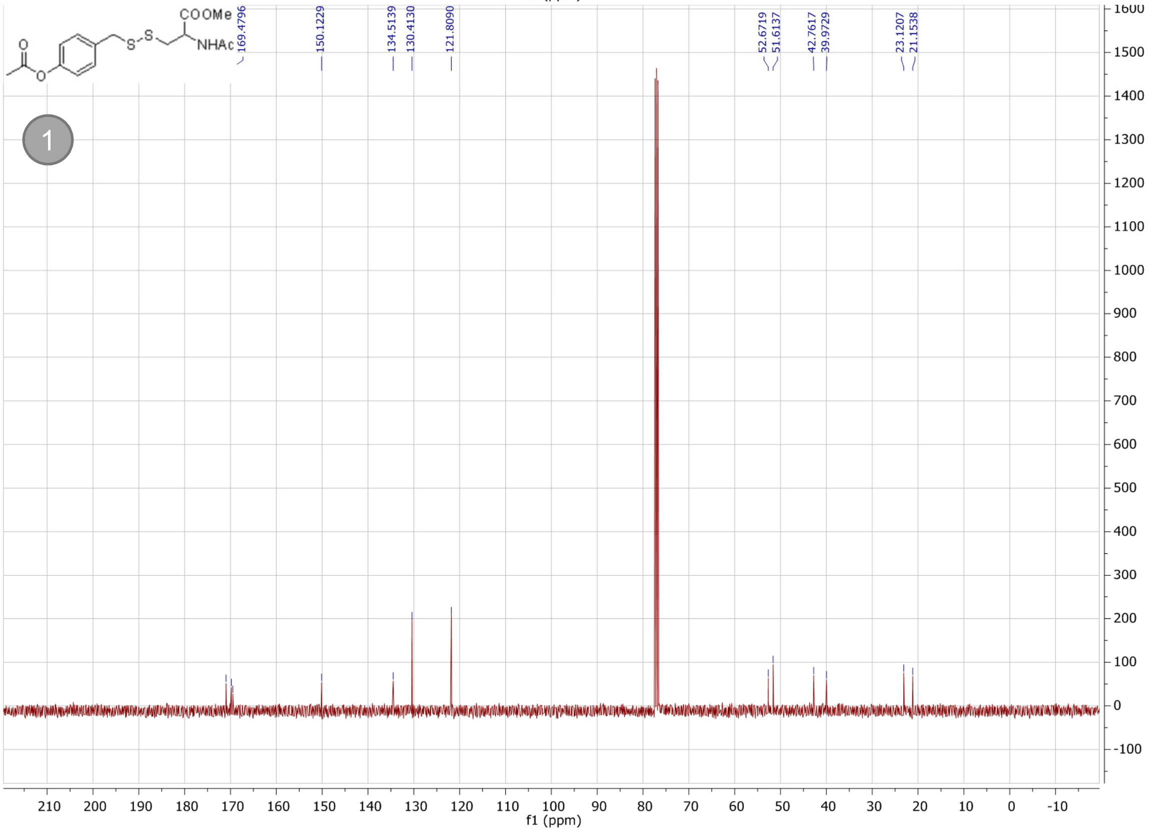
COOMe
NHAc
S
S
O
O
1
169.4796
150.1229
134.5139
130.4130
121.8090
52.6719
51.6137
42.7617
39.9729
23.1207
21.1538
f1 (ppm)

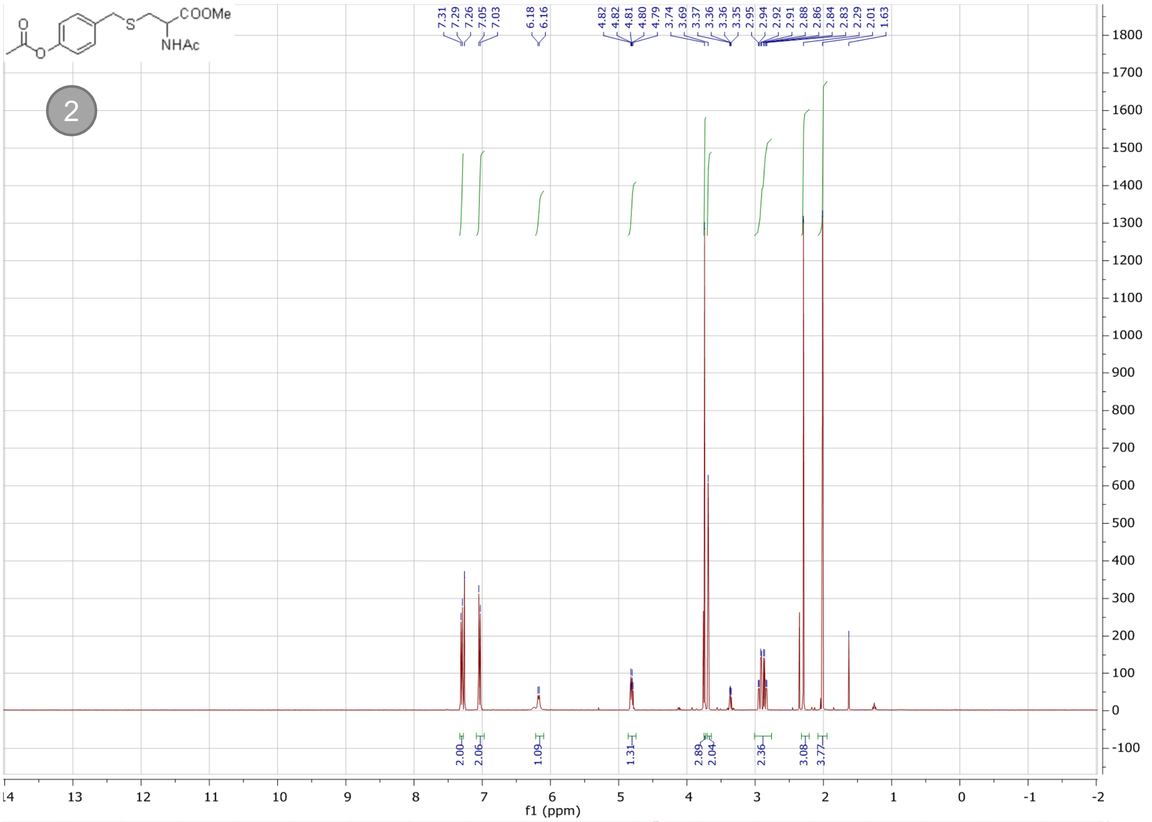
COOMe
S
O
O
NHAc
2
7.31
7.29
7.26
7.05
7.03
6.18
6.16
4.82
4.82
4.81
4.80
4.79
3.74
3.69
3.37
3.36
3.36
3.35
2.95
2.94
2.92
2.91
2.88
2.86
2.84
2.83
2.29
2.01
1.63
2.00
2.06
1.09
1.31
2.89
2.04
2.36
3.08
3.77
f1 (ppm)

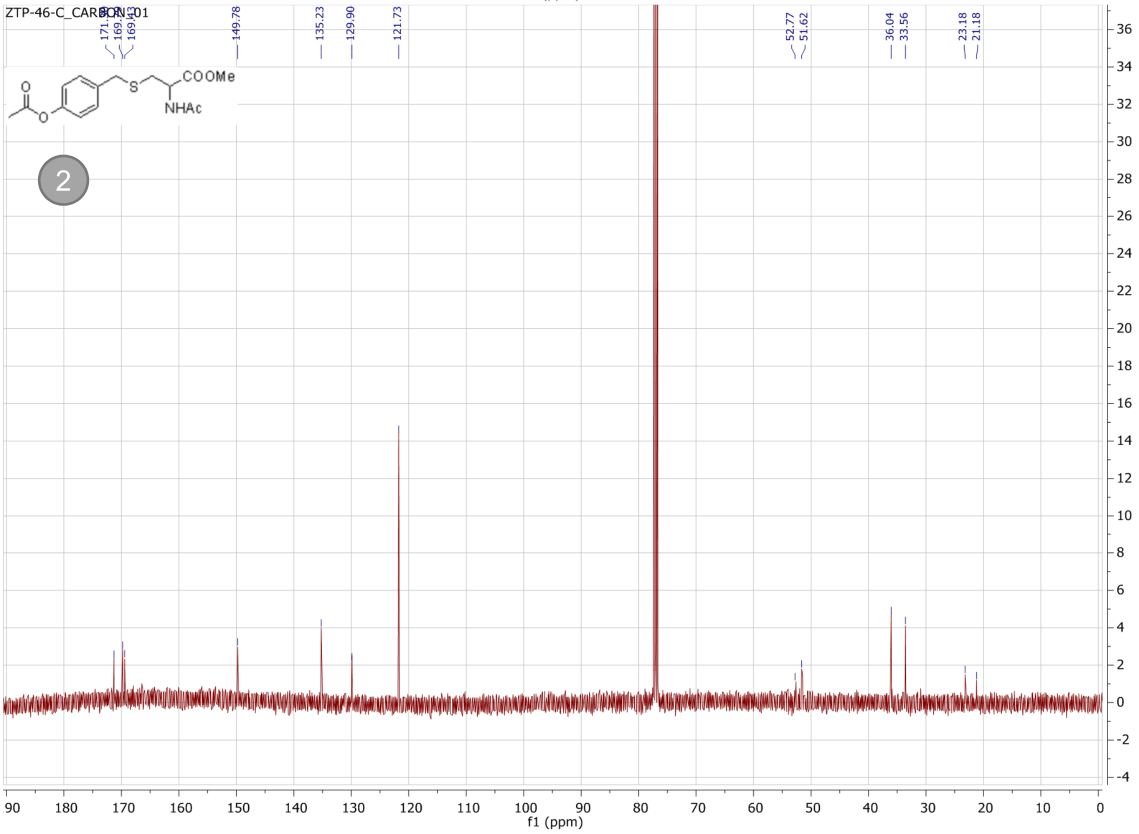
ZTP-46-C_CARBON_01
COOMe
S
O
O
NHAc
2
149.78
135.23
129.90
121.73
52.77
51.62
36.04
33.56
23.18
21.18
f1 (ppm)

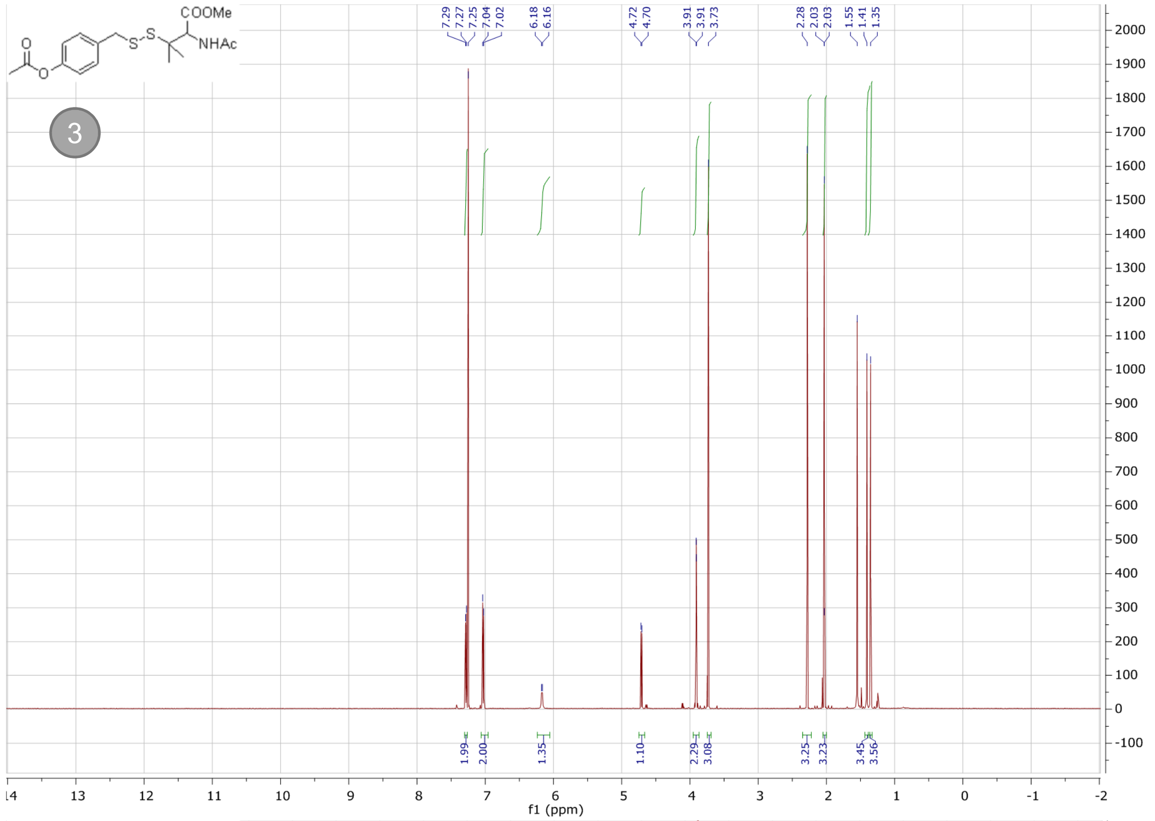
COOMe
NHAc
3
7.29 7.27 7.25 7.04 7.02 6.18 6.16 4.72 4.70 3.91 3.91 3.73 2.28 2.03 2.03 1.55 1.41 1.35
1.99 2.00 1.35 1.10 2.29 3.08 3.25 3.23 3.45 3.56
f1 (ppm)

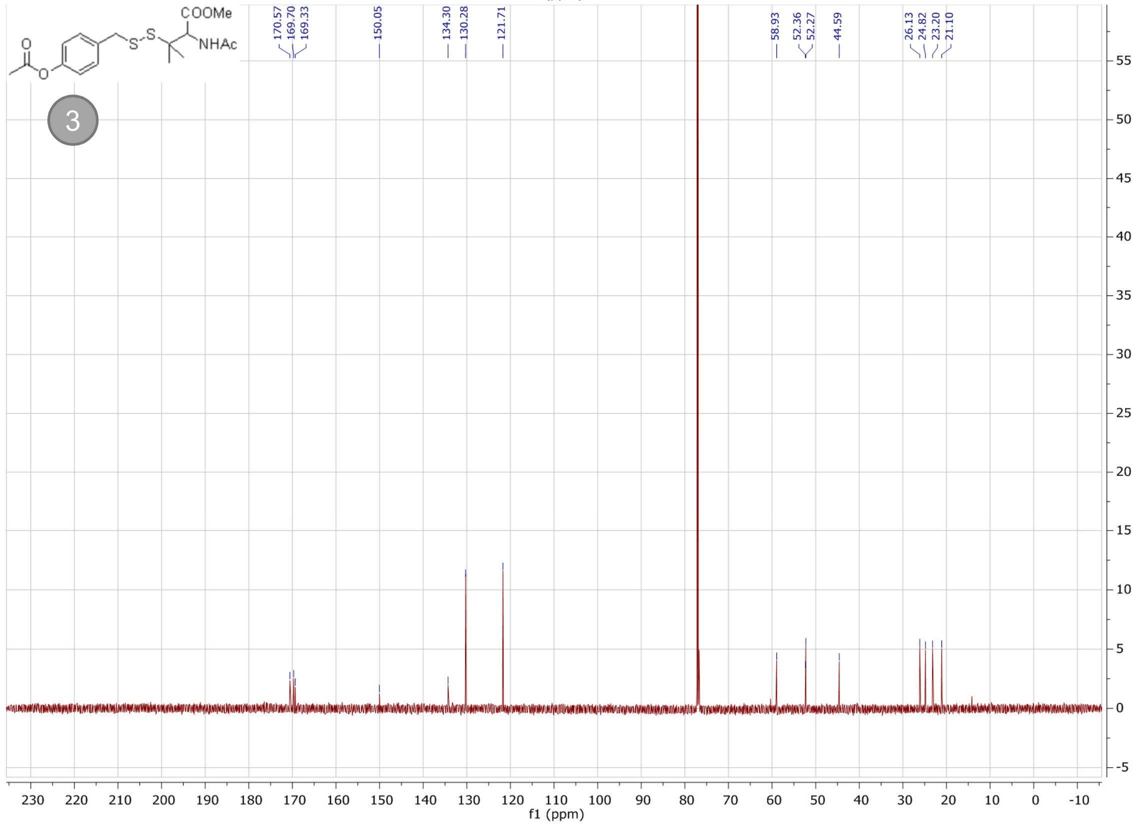
COOMe
NHAc
3
170.57 169.70 169.33 150.05 134.30 130.28 121.71 58.93 52.36 52.27 44.59 26.13 24.82 23.20 21.10
f1 (ppm)

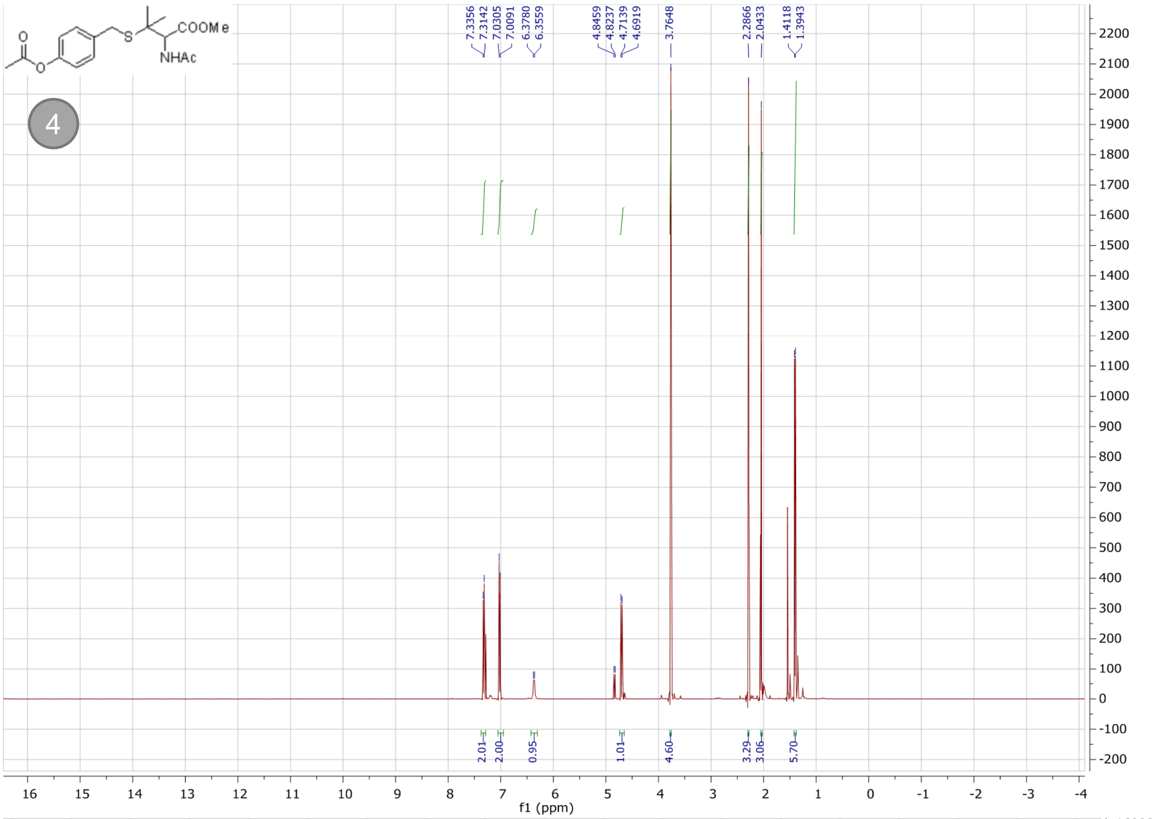
COOMe
S
NHAc
O
O
4
7.3356
7.3142
7.0305
7.0091
6.3780
6.3559
4.8459
4.8237
4.7139
4.6919
3.7648
2.2866
2.0433
1.4118
1.3943
2.01
2.00
0.95
1.01
4.60
3.29
3.06
5.70
f1 (ppm)

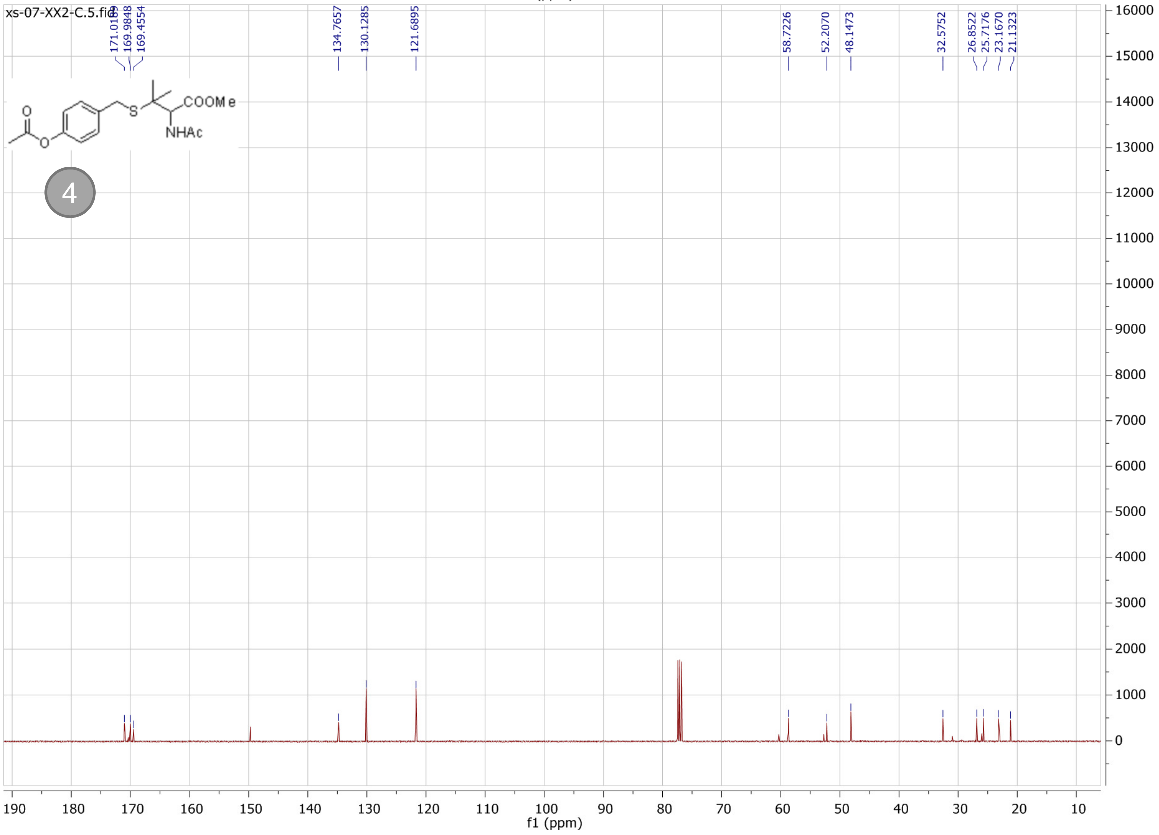
xs-07-XX2-C.5.fid
171.01
169.9848
169.4554
134.7657
130.1285
121.6895
58.7226
52.2070
48.1473
32.5752
26.8522
25.7176
23.1670
21.1323
COOMe
S
NHAc
O
O
4
f1 (ppm)

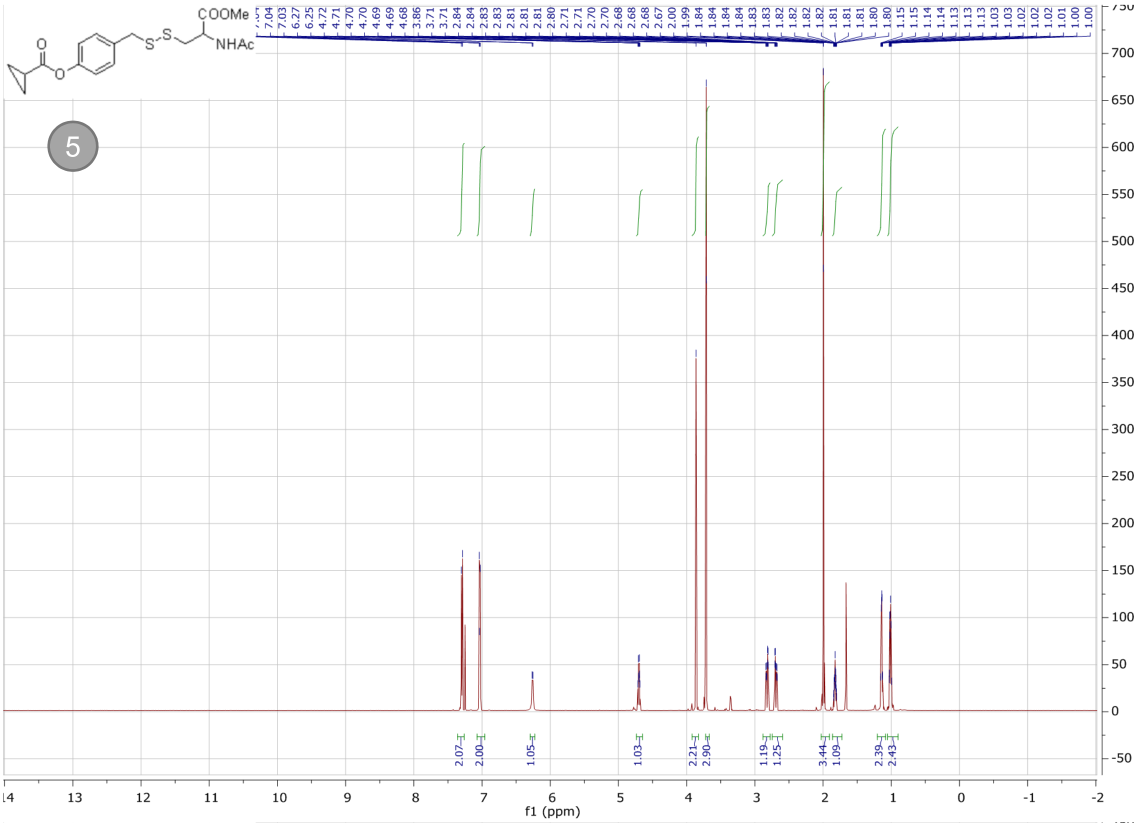
COOMe
NHAc
5
f1 (ppm)

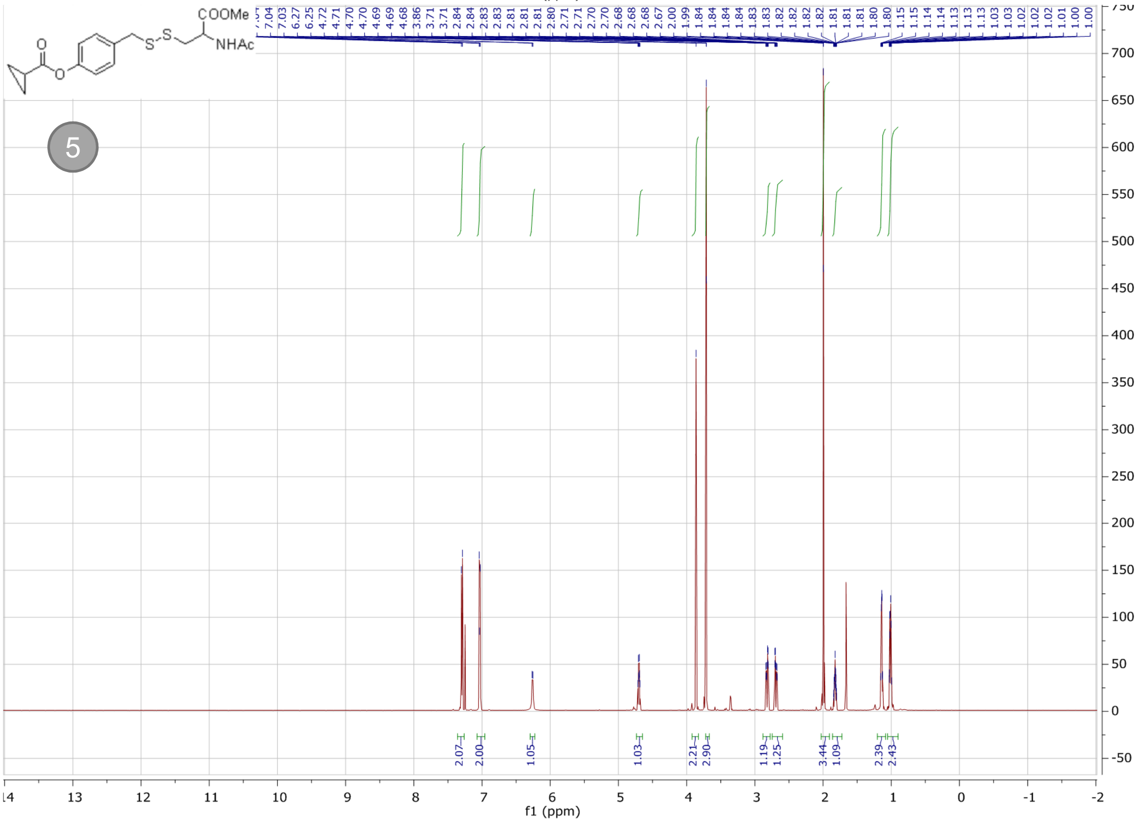
COOMe
NHAc
5
f1 (ppm)

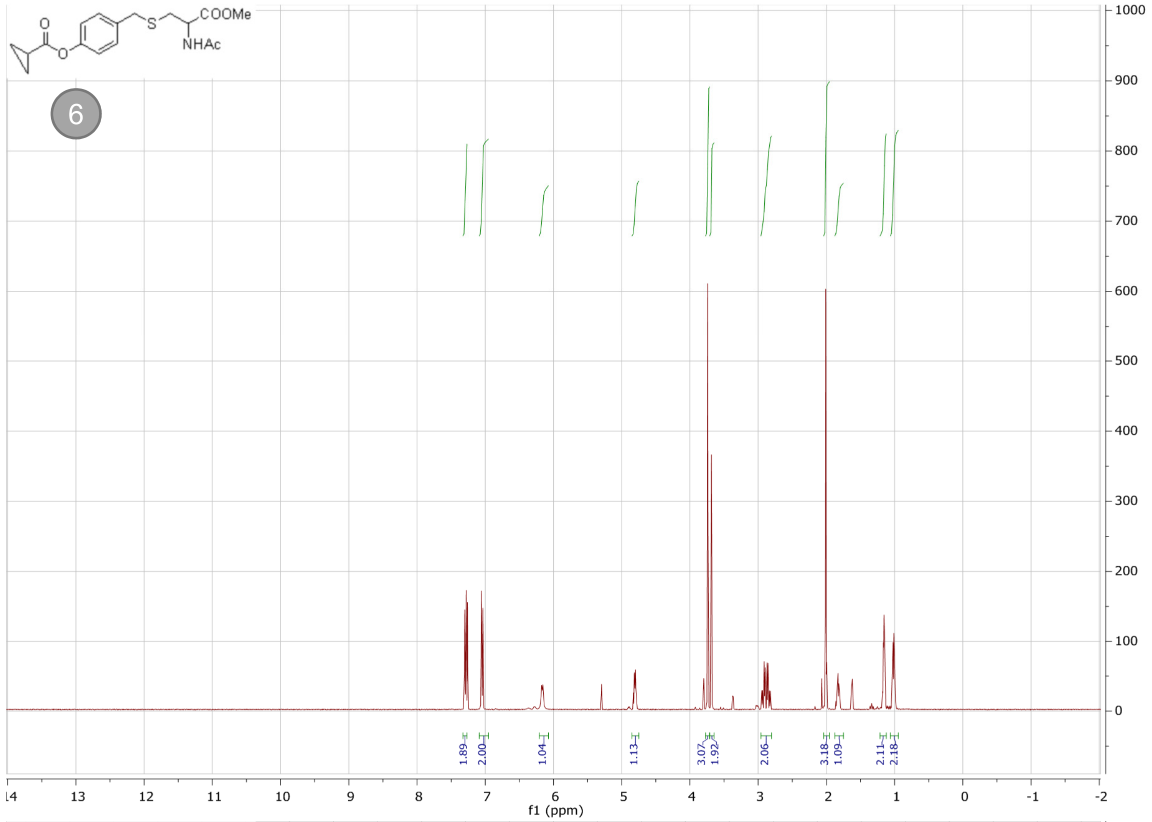
COOMe
NHAc
6
1.89
2.00
1.04
1.13
3.07
1.92
2.06
3.18
1.09
2.11
2.18
f1 (ppm)

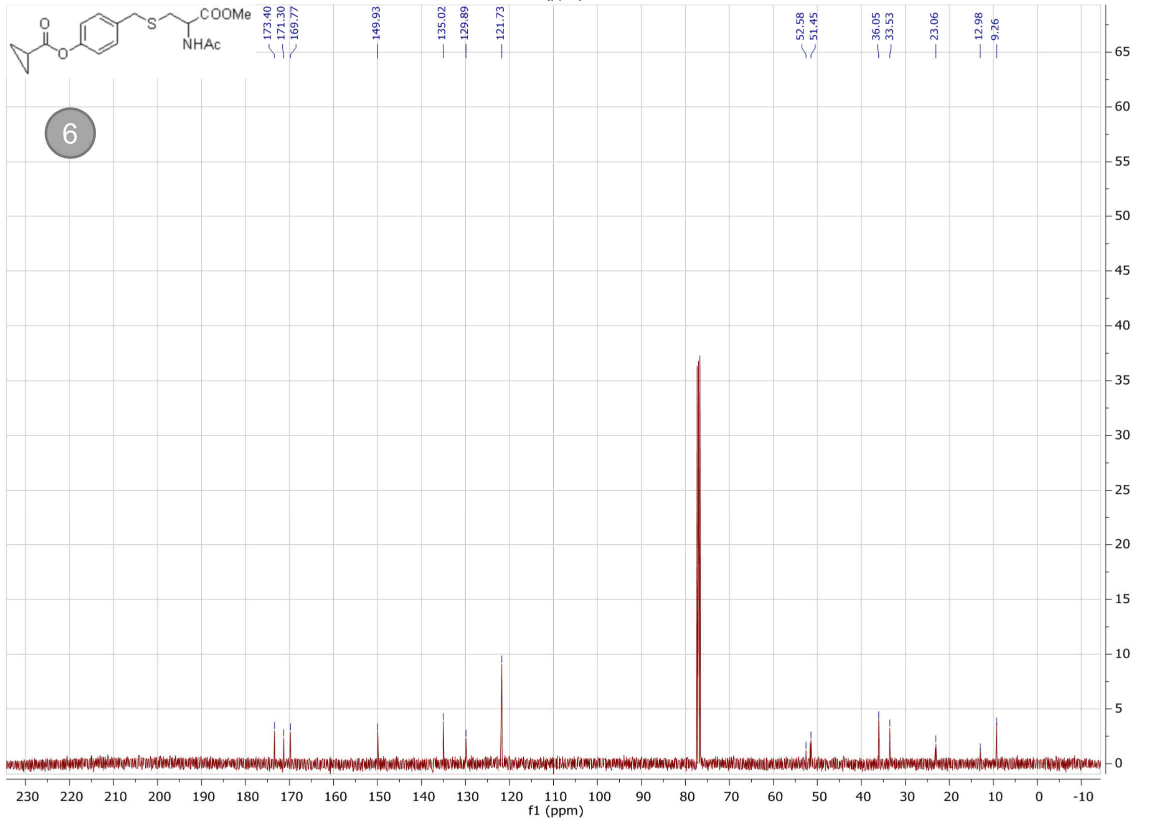
COOMe
NHAc
6
173.40
171.30
169.77
149.93
135.02
129.89
121.73
52.58
51.45
36.05
33.53
23.06
12.98
9.26
f1 (ppm)

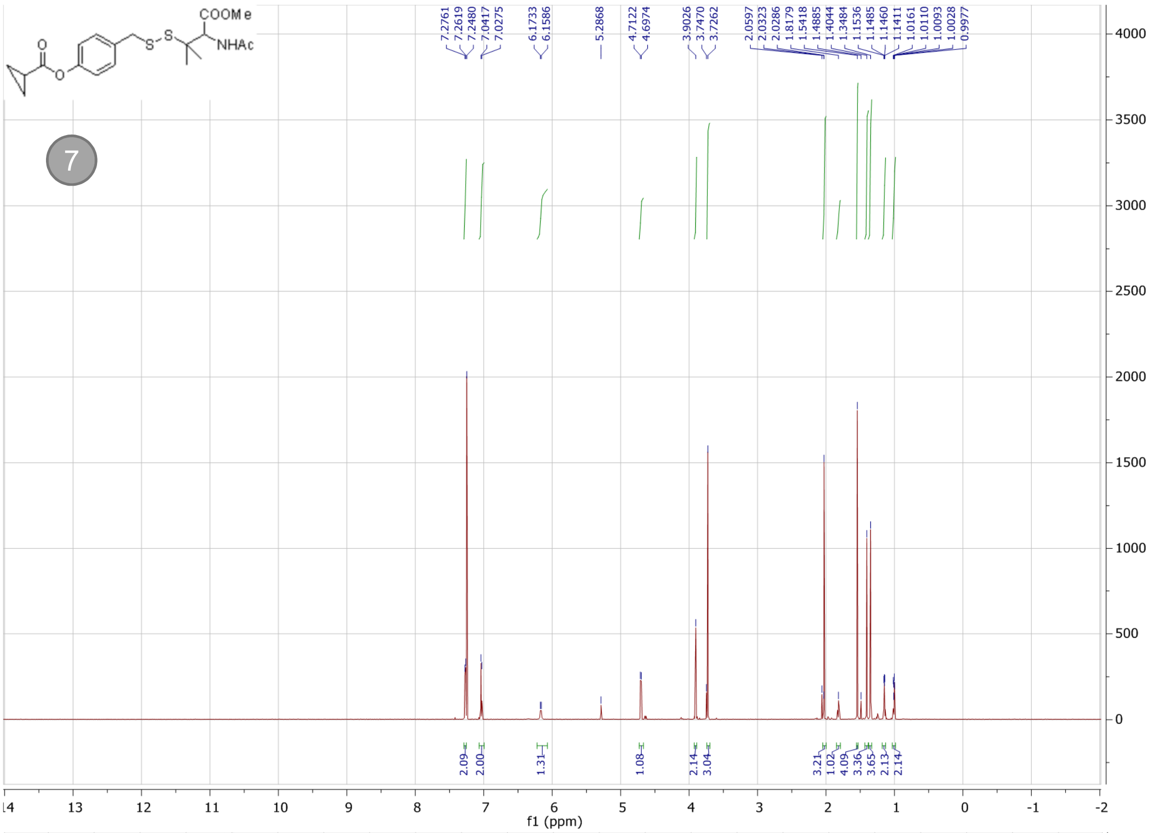
COOMe
NHAc
S
S
O
O
7
7.2761
7.2619
7.2480
7.0417
7.0275
6.1733
6.1586
5.2868
4.7122
4.6974
3.9026
3.7470
3.7262
2.0597
2.0323
2.0286
1.8179
1.5418
1.4885
1.4044
1.3484
1.1536
1.1485
1.1460
1.1411
1.0161
1.0110
1.0093
1.0028
0.9977
2.09
2.00
1.31
1.08
2.14
3.04
3.21
1.02
4.09
3.36
3.65
2.13
2.14
f1 (ppm)

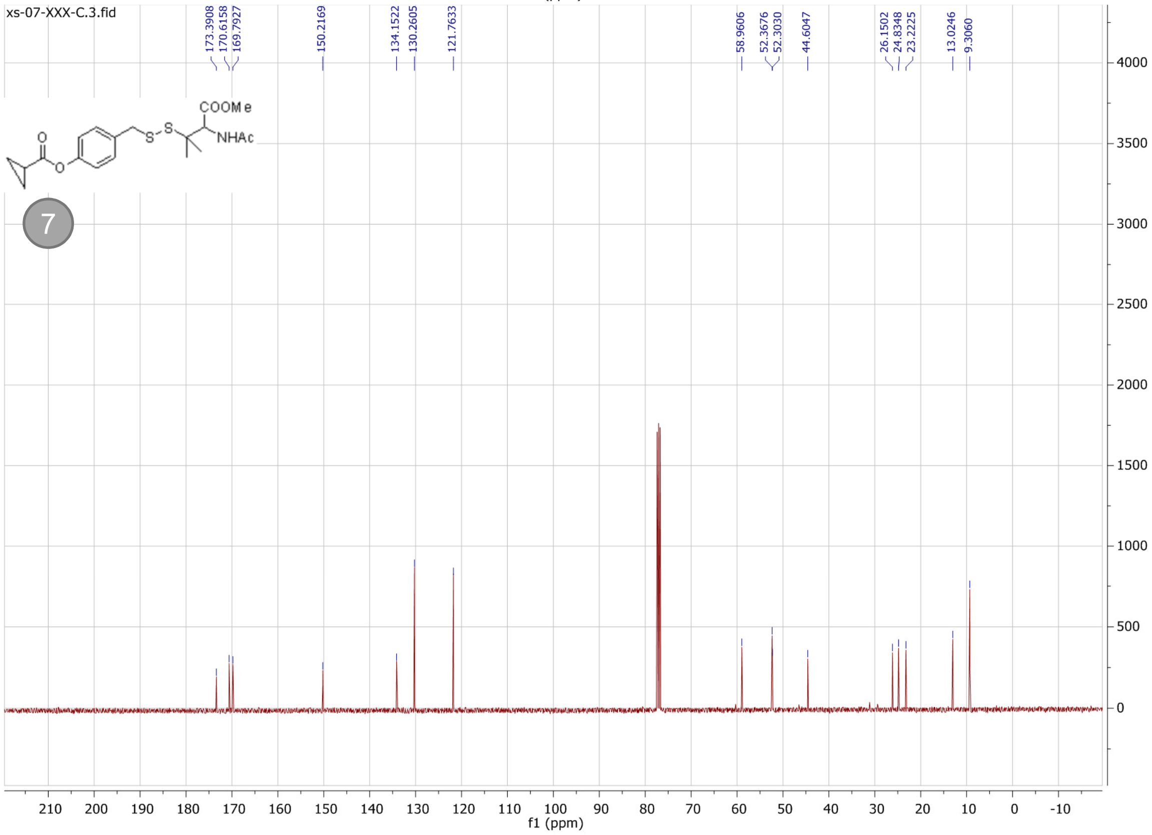
xs-07-XXX-C.3.fid
173.3908
170.6158
169.7927
150.2169
134.1522
130.2605
121.7633
58.9606
52.3676
52.3030
44.6047
26.1502
24.8348
23.2225
13.0246
9.3060
COOMe
NHAc
S
S
O
O
7
f1 (ppm)

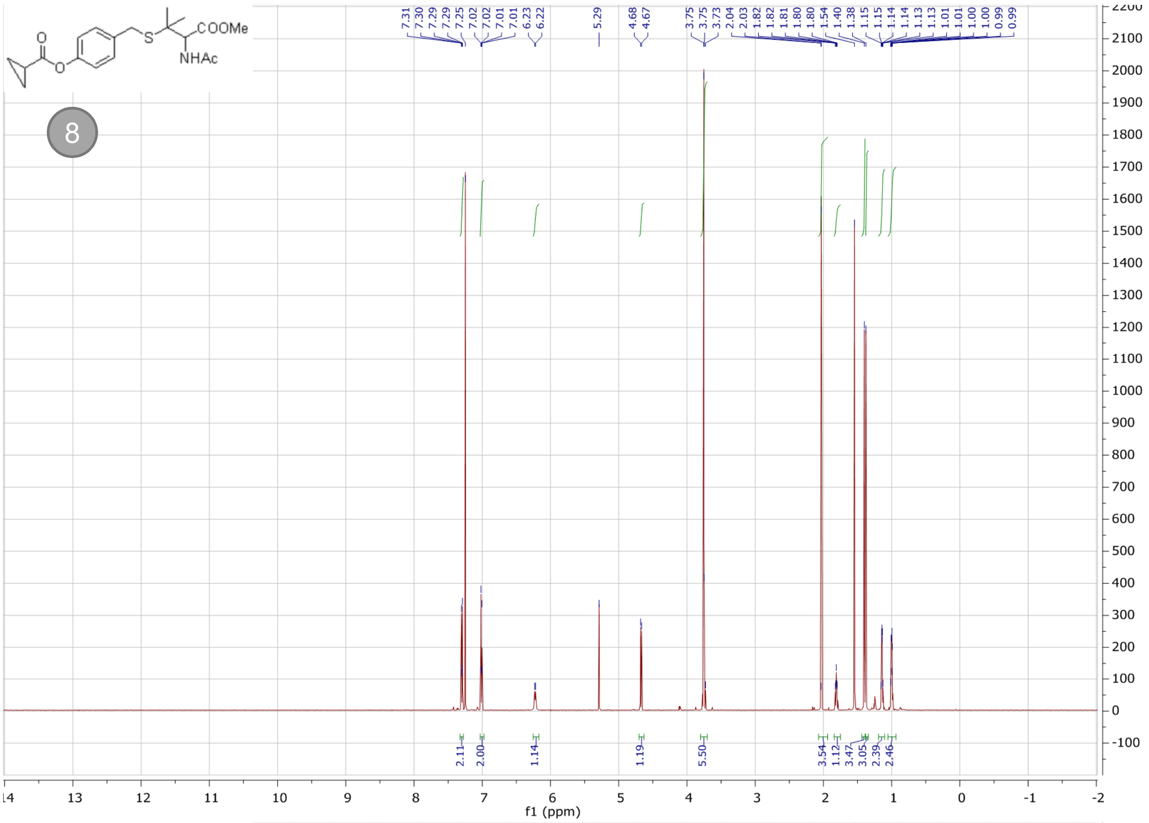

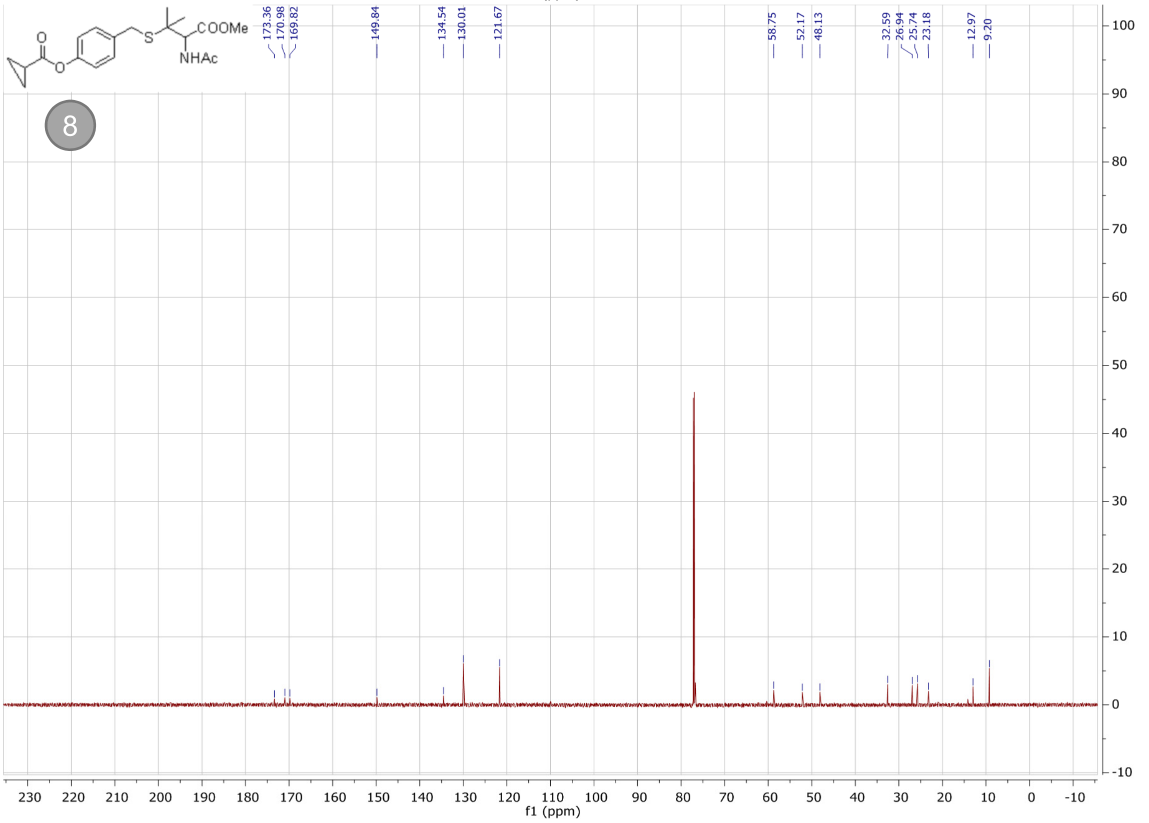

Supplementary Figure 3 Analysis of controllable sulfane sulfur donors (1, 3 5, and 7) and their control compounds (2, 4 6, and 8) using ~~MS~~ spectropscopy
